# Optogenetic manipulation of Gq- and Gi/o-coupled receptor signaling in neurons and heart muscle cells

**DOI:** 10.7554/eLife.83974

**Published:** 2023-08-17

**Authors:** Hanako Hagio, Wataru Koyama, Shiori Hosaka, Aysenur Deniz Song, Janchiv Narantsatsral, Koji Matsuda, Tomohiro Sugihara, Takashi Shimizu, Mitsumasa Koyanagi, Akihisa Terakita, Masahiko Hibi

**Affiliations:** 1 Graduate School of Science, Nagoya University Nagoya Japan; 2 Graduate School of Bioagricultural Sciences, Nagoya University Nagoya Japan; 3 Institute for Advanced Research, Nagoya University Nagoya Japan; 4 Graduate School of Science, Osaka Metropolitan University Osaka Japan; Nanyang Technological University Singapore; Max Planck Institute for Heart and Lung Research Germany

**Keywords:** optogenetics, bistable rhodopsin, g protein-coupled rhodopsin, g protein-coupled receptor, locomotion, cardiac contraction, Zebrafish

## Abstract

G-protein-coupled receptors (GPCRs) transmit signals into cells depending on the G protein type. To analyze the functions of GPCR signaling, we assessed the effectiveness of animal G-protein-coupled bistable rhodopsins that can be controlled into active and inactive states by light application using zebrafish. We expressed Gq- and Gi/o-coupled bistable rhodopsins in hindbrain reticulospinal V2a neurons, which are involved in locomotion, or in cardiomyocytes. Light stimulation of the reticulospinal V2a neurons expressing Gq-coupled spider Rh1 resulted in an increase in the intracellular Ca^2+^ level and evoked swimming behavior. Light stimulation of cardiomyocytes expressing the Gi/o-coupled mosquito Opn3, pufferfish TMT opsin, or lamprey parapinopsin induced cardiac arrest, and the effect was suppressed by treatment with pertussis toxin or barium, suggesting that Gi/o-dependent regulation of inward-rectifier K^+^ channels controls cardiac function. These data indicate that these rhodopsins are useful for optogenetic control of GPCR-mediated signaling in zebrafish neurons and cardiomyocytes.

## Introduction

G-protein-coupled receptors (GPCRs) are responsible for transmitting extracellular signals into the cell. Many of them function as receptors for neurotransmitters or hormones, and activate coupled trimeric G proteins consisting of α, β, and γ subunits (Hilger et al., 2018; Pierce et al., 2002; Rockman et al., 2002; Rosenbaum et al., 2009). Upon activation of a GPCR, the α subunit (Gα) is converted from a GDP- to a GTP-bound form to regulate target proteins, while β and γ subunits are released from Gα as a complex (Gβγ) to control their own target proteins. GPCR regulates different signaling cascades depending on the type of Gα that they bind (e.g. Gs, Gq, Gt, and Gi/o). Gs- and Gi/o-coupled GPCRs activate and inhibit, respectively cAMP-producing adenylyl cyclase (AC) via the Gα subunits. Gi/o-coupled GPCRs also regulate G protein activated inward-rectifier K^+^ channels (GIRKs) via the Gβγ subunit, increasing K^+^ efflux and thereby inducing hyperpolarization. In contrast, Gq-coupled GPCRs, via their Gα subunits, activate phospholipase β (PLCβ) to generate inositol 1,4,5-triphosphate (IP3) and diacylglycerol (DAG) from phosphatidyl 4,5-bisphosphate (PIP2), subsequently elevating intracellular Ca^2+^ and activating protein kinase C (PKC). For example, in the central nervous system, the neurotransmitter glutamate binds to and activates GPCRs that are referred to as metabotropic receptors (mGluRs), some of which function as Gq-coupled GPCRs (e.g. mGluR1), and others as Gi/o-coupled GPCRs (e.g. mGluR2, 3; Reiner and Levitz, 2018). In the heart, noradrenaline binds to and activates the Gs-coupled β1 adrenergic receptor (β1AR), which increases myocardial contraction and heart rate (de Lucia et al., 2018), while acetylcholine binds to and activates the Gi/o-coupled muscarinic M2 receptor, which reduces heart rate and contraction (Wess et al., 2007). Although the functions of many GPCR signals have been studied, exactly in which cells, when, and how they function have not yet been fully elucidated. To solve these unknowns, it is necessary to precisely manipulate the location and timing of GPCR signaling.

Several techniques have been developed to control the activity and signaling of target cells. Chemogenetics using artificially designed GPCRs that are derived from muscarinic M3 receptor and can be activated by chemical ligands (Designer Receptor Exclusively Activated by Designer Drugs, DREADD) (Armbruster et al., 2007; Kaganman, 2007; Roth, 2016; Wess et al., 2013) has been used to control GPCR signaling, but achieving temporally and spatially precise control has been difficult. In contrast, optogenetics using rhodopsins, which bind to a chromophore retinal and can regulate their function in a light-sensitive manner, has been used to control and study cell functions. Light-gated microbial channelrhodopsins (e.g. ChR2) and light-driven microbial ion pump-type rhodopsins (e.g. halorhodopsin, NpHR) have been exploited to control the activities of neurons and/or cardiomyocytes (Arrenberg et al., 2009; Arrenberg et al., 2010; Boyden et al., 2005; Deisseroth and Hegemann, 2017). However, these rhodopsins induce depolarization or hyperpolarization of the membrane potential of cells in a light stimulus-dependent manner at a precise timing and locations, but do not directly control GPCR signaling. In contrast, animal rhodopsins are light-activated G-protein-coupled proteins and can activate various signaling cascades, like GPCRs for neurotransmitters and hormones, while displaying a diversity of wavelength sensitivity and G-protein selectivity (Koyanagi et al., 2021; Koyanagi and Terakita, 2014; Terakita, 2005).

Most animal rhodopsins bind to 11-*cis* retinal, which is isomerized to an all-*trans* form upon light absorption. This isomerization triggers a conformational change of rhodopsins and activates signal transduction cascades via the coupled G protein. Vertebrate visual rhodopsins release the chromophore all-*trans* retinal after light absorption and become an inactive form (bleach). The photoregeneration of these rhodopsins depends on the enzymes that generate 11-*cis* retinal, such as retinal isomerases, which are specifically expressed in photoreceptor organs (Koyanagi et al., 2021; Koyanagi and Terakita, 2014; Terakita, 2005; Terakita et al., 2015). Therefore, the photosensitivity of visual rhodopsins might not be very stable in cells other than photoreceptor organs. In contrast, animal rhodopsins other than vertebrate visual rhodopsins retain 11-*cis* retinal and convert into photoproducts having the all-*trans* form (active state) upon light absorption, and these then revert to the original (inactive) dark state by subsequent light absorption, so they are bleach-resistant and are called bistable opsins (Koyanagi et al., 2005; Koyanagi et al., 2021; Koyanagi and Terakita, 2014; Terakita and Nagata, 2014; Terakita et al., 2015; Tsukamoto and Terakita, 2010; Tsukamoto et al., 2005). Although chimeric optogenetic tools have been engineered using visual opsins with cytoplasmic loops and the C-terminal tail of adrenergic receptors (Opto-XRs) (Airan et al., 2009; Kim et al., 2005; Siuda et al., 2015; Spangler and Bruchas, 2017), bistable opsins have the advantage of stable optical control of GPCR signaling in various tissues.

A number of Gq-coupled bistable rhodopsin families have been identified as visual opsins in arthropods and molluscs, and as melanopsin in both vertebrates and invertebrates (Koyanagi and Terakita, 2008). Among them, jumping spider rhodopsin-1 (SpiRh1) was isolated from the jumping spider *Hasarius adansoni* and was reported to activate the Gq-signaling cascade in a green light-dependent manner (Koyanagi et al., 2008; Nagata et al., 2012). Mosquito Opn3 (MosOpn3) is an invertebrate homolog of vertebrate Opn3 (Hill et al., 2002). The Opn3 group contains multiple members including Opn3, originally called encephalopsin, teleost multiple tissue (TMT) opsin, etc. (Blackshaw and Snyder, 1999; Koyanagi et al., 2021; Moutsaki et al., 2003; Terakita, 2005; Terakita and Nagata, 2014). When MosOpn3 was expressed in mammalian cultured cells, it bound to both 11-*cis* and 13-*cis* retinal (Koyanagi et al., 2013). MosOpn3 light-dependently activated Gi- and Go-type G proteins in vitro and initiated a Gi-signaling cascade in cultured cells (Koyanagi et al., 2013). Parapinopsin, which belongs to another group of bistable opsins, serves as a Gt-coupled opsin, like vertebrate visual opsins, and can also activate Gi-type G protein in vitro and in mammalian cultured cells (Kawano-Yamashita et al., 2015; Koyanagi et al., 2021; Terakita et al., 2004; Tsukamoto et al., 2009). The stable photoproduct (active form) of parapinopsin has its absorption maximum at ~500 nm, which is considerably distant from that of the dark state (~360 nm). Therefore, light illumination with different wavelengths was shown to switch on and off G-protein-mediated signaling via parapinopsin in vitro and in cultured cells (Kawano-Yamashita et al., 2015; Koyanagi et al., 2004; Wada et al., 2018). MosOpn3 and lamprey parapinopsin (LamPP) were used to suppress neuronal activities in a light stimulation-dependent manner in mammals (Copits et al., 2021; Mahn et al., 2021; Rodgers et al., 2021) and in *Caenorhabditis elegans* (Koyanagi et al., 2022). In addition to these rhodopsins, a Gq-coupled rhodopsin, neuropsin (also known as Opn5), was used to induce the activation of neurons, the intestine, and heart (Dai et al., 2022; Wagdi et al., 2022). Optogenetic activation of Gs-coupled rhodopsin jellyfish opsin (JellyOp) and Gi-coupled long wavelength-sensitive cone opsin (LWO) were shown to accelerate and suppress the excitation of cardiomyocytes, respectively (Cokić et al., 2021; Makowka et al., 2019). However, it remains unclear whether they can control GPCR signaling in other types of cells and what mechanisms underlie optogenetic controls of GPCR signaling in each cell type. Zebrafish larvae (especially pigment-deficient mutants) are transparent, so zebrafish have been used for analyses using optogenetic tools (Antinucci et al., 2020; Arrenberg et al., 2010; Bernal Sierra et al., 2018; Umeda et al., 2013). In this study, we examined the optogenetic activity of Gq- and Gi/o-coupled animal bistable rhodopsins (listed in Table 1) by expressing them in hindbrain reticulospinal V2a neurons that drive locomotion and cardiomyocytes in zebrafish.

## Results

### Activity of G-protein-coupled bistable rhodopsins in human cells

**Figure 1.**
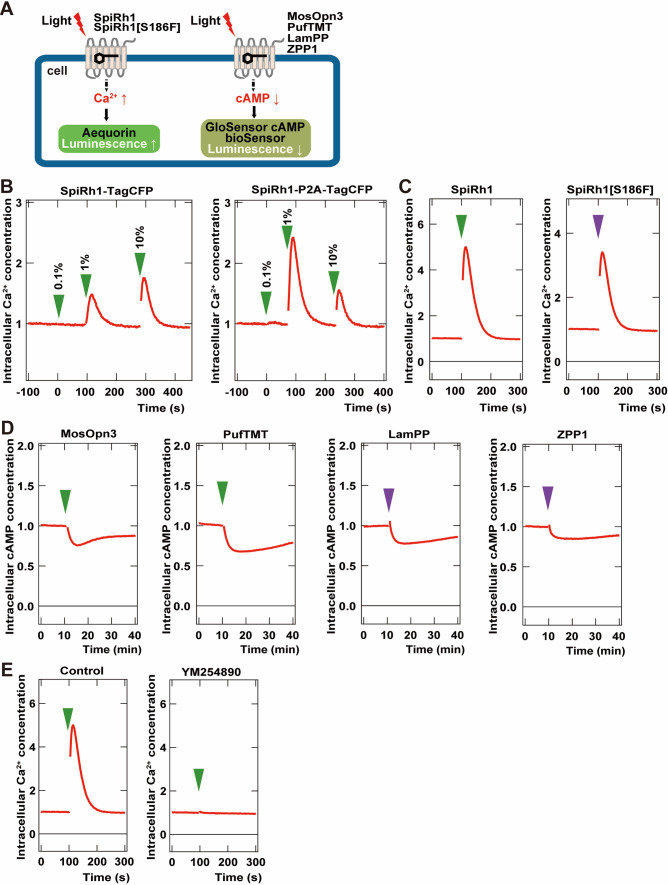
Activity of G-coupled bistable rhodopsins in HEK293S cells. (**A**) Schematic of intracellular Ca^2+^ or cAMP measurements in HEK293S cells. Intracellular Ca^2+^ and cAMP concentrations in rhodopsin-expressing HEK293S cells were measured using the aequorin m2 assay or the GloSensor 20 F assay, respectively. (**B**) Comparison of optogenetic activities of Gq-coupled Spider Rh1 (SpiRh1) expressed using TagCFP fusion protein and the P2A-TagCFP system. HEK293S cells were transfected with an expression plasmid for the fusion protein of SpiRh1 and TagCFP (SpiRh1-TagCFP, left panel), or for that of Flag-tagged SpiRh1, porcine teschovirus 2 A peptide, and TagCFP (SpiRh1-P2A-TagCFP, right panel). Transfected cells were incubated with 11-*cis* retinal and stimulated by different intensities of 500 nm light (0.1%, 1%, or 10% of the light intensity, with 0.106 mW/mm^2^ as 100%). Intracellular Ca^2+^ concentration was measured by using aequorin m2 and is indicated as a ratio to the unstimulated state in the graphs. (**C**) Comparison of activities of Flag-tagged SpiRh1 and SpiRh1 [S186F]. Transfected cells were stimulated by 500 nm (green arrow, 0.106 mW/mm^2^) or 410 nm (purple arrow, 0.0194 mW/mm^2^) light and intracellular Ca^2+^ concentration was measured. (**D**) Light-stimulus-dependent reduction of intracellular cAMP level by Gi/o-coupled mosquito Opn3 (MosOpn3), pufferfish TMT (PufTMT), lamprey PP (LamPP), and zebrafish PP1 (ZPP1). HEK293S cells were transfected with expression plasmids for flagged-tagged Gi/o rhodopsins. Transfected cells were incubated with 11-*cis* retinal and stimulated by 500 nm (green arrow) or 410 nm (purple arrow) light. Intracellular cAMP concentration was measured with GloSensor 20 F and is indicated as a ratio to the unstimulated state. (**E**) Effects of Gαq inhibitor YM254890 on SpiRh1. HEK293S cells transfected with an expression plasmid for SpiRh1 were incubated with 11-*cis* retinal alone (left panel) or with 11-*cis* retinal and YM254890 (right panel), and stimulated by 500 nm light. Intracellular Ca^2+^ concentration was measured. Figure 1—source data 1.Data for Figure 1, activity of bistable rhodopsins in HEK293S cells.

To examine the activity of G-protein-coupled rhodopsins in cells, we created two DNA constructs that expressed a rhodopsin and a fluorescent protein as a fusion protein, or that expressed a carboxy-terminal Flag epitope-tagged rhodopsin and a fluorescent protein separately using a viral 2 A (P2A) peptide system. We first expressed a fusion protein of Gq-coupled SpiRh1 (Koyanagi et al., 2008; Nagata et al., 2012) and TagCFP (SpiRh1-TagCFP), or Flag-tagged SpiRh1 and TagCFP separately (SpiRh1-P2A-TagCFP), in human embryonic kidney 293S (HEK293S) cells. The effect of photoactivation of these proteins on intracellular Ca^2+^ level was examined by the aequorin assay (Bailes and Lucas, 2013; Figure 1A). Light stimulation increased intracellular Ca^2+^ at a much higher level for SpiRh1-P2A-TagCFP-expressing cells than SpiRh1-TagCFP-expressing cells, suggesting that the expression level and/or activity of bistable rhodopsins is higher with a Flag epitope-tagged protein than with a large fluorescent-fused protein (Figure 1B). The light stimulation-dependent increase in intracellular Ca^2+^ with SpiRh1 was suppressed by treatment with a Gαq inhibitor YM254890 (Figure 1E), confirming that SpiRh1 mediates Gq-mediated signaling. We created similar Flag-tagged expression constructs for bistable Gq- and Gi/o-coupled rhodopsins from various invertebrate and vertebrate animals listed in Table 1 and expressed these rhodopsins in HEK293S cells. The effect of photoactivation of these rhodopsins on intracellular Ca^2+^ or cAMP level was examined by the aequorin and noi GloSensor cAMP assays (Bailes and Lucas, 2013; Figure 1A). These included Gq-coupled SpiRh1[S186F], a SpiRh1 mutant that has a maximal sensitivity in the UV region (Nagata et al., 2019) as well as Gi/o-coupled MosOpn3 (carboxy-terminal truncated MosOpn3 was used) (Koyanagi et al., 2013), pufferfish TMT opsin (PufTMT) (Koyanagi et al., 2013), LamPP (Koyanagi et al., 2004), and zebrafish parapinopsin1 (ZPP1) (Koyanagi et al., 2015). Stimulation of SpiRh1- and SpiRh1[S186F]-expressing cells with 500 and 410 nm light, respectively, increased intracellular Ca^2+^ (Figure 1C). Light stimulation of cells expressing MosOpn3, PufTMT, LamPP, or ZPP1 with 500 (for MosOpn3 and PufTMT) or 410 nm (for LamPP and ZPP1) light reduced intracellular cAMP levels to similar extents (Figure 1D). These data indicate that these Flag-tagged G-protein-coupled rhodopsins can be used for optogenetic manipulation of Gq- and Gi/o-mediated signaling in human HEK293S cells.

### Optogenetic activation of zebrafish locomotion circuit by Spider Rh1

**Figure 2.**
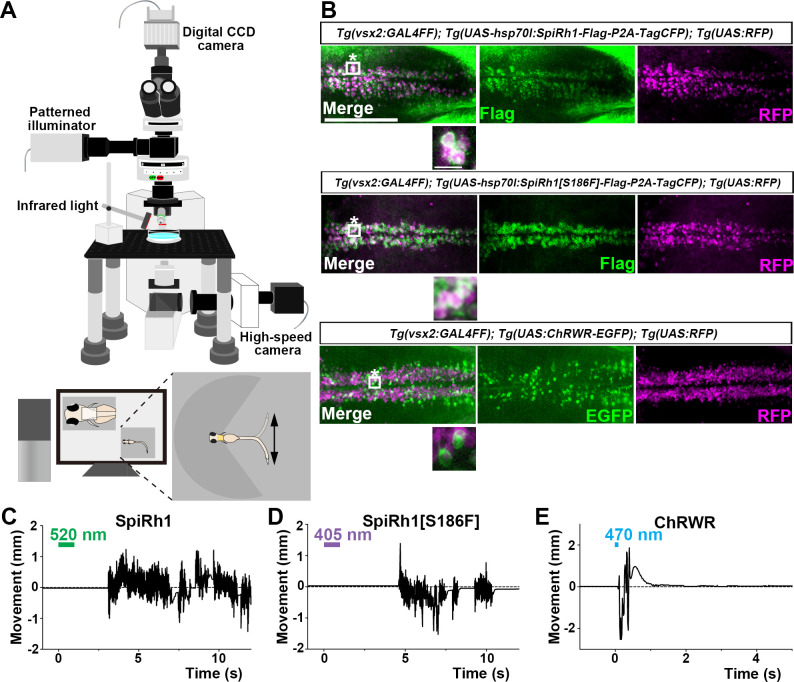
Activation of reticulospinal V2a neurons by Gq-coupled bistable rhodopsins. (**A**) Schematic of experimental devices for induction of swimming behavior and a larva embedded in agarose. The hindbrain region was irradiated with light by using a patterned illuminator. Tail (caudal fin) movements were monitored by a high-speed camera with infrared light. (**B**) Expression of SpiRh1, SpiRh1[S186F], and channel rhodopsin wide receiver (ChRWR) in hindbrain reticulospinal V2a neurons. 3-dpf (days post fertilization) *Tg(vsx2:GAL4FF);Tg(UAS-hsp70l:SpiRh1-Flag-P2A-TagCFP, myl7:mCherry);Tg(UAS:RFP), Tg(vsx2:GAL4FF);Tg(UAS-hsp70l:SpiRh1[S186F]-Flag-P2A-TagCFP, myl7:mCherry);Tg(UAS:RFP*) and *Tg(vsx2:GAL4FF);Tg(UAS:ChRWR-EGFP);Tg(UAS:RFP*) larvae were fixed and stained with anti-Flag or anti-GFP (EGFP, green), and anti-DsRed (RFP, magenta) antibodies. Inset: higher magnification views of the boxed areas showing double-labeled neurons. (**C, D, E**) Tail movements of 3-dpf Tg larvae expressing SpiRh1 (**C**), SpiRh1 [S186F] (**D**), and ChRWR (**E**) in the reticulospinal V2a neurons after light stimulation. The hindbrain area was stimulated with light (0.4 mW/mm^2^) of wavelengths of 520 nm (for SpiRh1), 405 nm (for SpiRh1[S186F]), and 470 nm (for ChRWR) for 1 s (for SpiRh1 and SpiRh1[S186F]) or 100 ms (for ChRWR). Typical movies are shown in Figure 2—videos 1–3. Scale bar = 150 μm in (**B**), 10 μm in the insets of (**B**). Figure 2—source data 1.Data for Figure 2C–E, tail movements of Tg larvae expressing SpiRh1, SpiRh1[S186F], and ChRWR.

**Figure 3.**
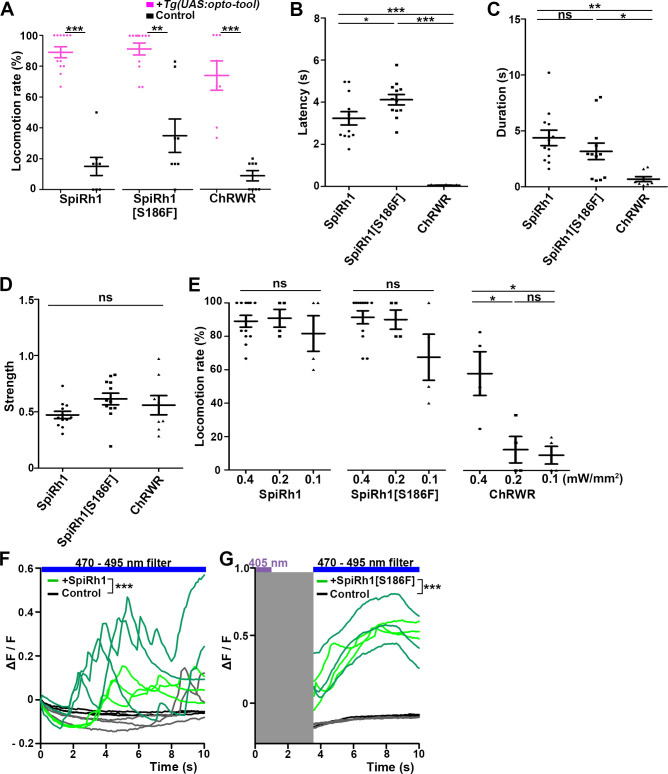
Locomotion induced by SpiRh1, SpiRh1[S186F], and ChRWR. (**A**) Light stimulus-dependent locomotion rates of 3-dpf Tg larvae expressing SpiRh1, SpiRh1[S186F] and ChRWR in hindbrain reticulospinal V2a neurons. Sibling larvae that did not express the tools were used as controls. The hindbrain area of the larvae was irradiated with light (0.4 mW/mm^2^) of wavelengths of 520 nm for 1 s (SpiRh1), 405 nm for 1 s (SpiRh1[S186F]), and 470 nm for 100 ms (ChRWR). Six consecutive stimulus trials were analyzed for 8 or 12 larvae of each Tg line (n=12 for SpiRh1 and SpiRh1[S186F], n=8 for ChRWR). For each larva, the average percentage of trials in which tail movement was elicited was calculated as the locomotion rates and plotted in graphs. Wilcoxon rank sum test (SpiRh1 vs control, p=0.000192; SpiRh1[S186F] vs control, p=0.00664; ChRWR vs control, p=0.000792). (**B, C, D**) Light stimulus-evoked tail movements of latency (**B**), duration (**C**), and strength (**D**). The time from the start of light application to the first tail movement was defined as latency (**s**), and the time from the beginning to the end of the first tail movement was defined as duration (**s**). The maximum distance the caudal fin moved from the midline divided by body length was measured as strength. One-way ANOVA with Tukey’s post hoc test (latency SpiRh1 vs SpiRh1[S186F], p=0.0424; SpiRh1 vs ChRWR, p=1.58e-08; SpiRh1[S186F] vs ChRWR, p=7.40 e-11; duration SpiRh1 vs ChRWR; p=0.00245; SpiRh1[S186F] vs ChRWR; p=0.0469). (**E**) Locomotion rates evoked by the stimulus light of various intensities. For each Tg line and each condition, six consecutive stimulus trials were analyzed for 4 or 12 larvae (n=12 for 0.4 mW/mm^2^ light stimulation with SpiRh1 and SpiRh1[S186F], n=4 for others) and the average locomotion rates were calculated. For comparison, Tg fish expressing ChRWR were also irradiated for 1 s. One-way ANOVA with Tukey’s post hoc test (ChRWR 0.4 mW/mm^2^ vs 0.2 mW/mm^2^, p=0.0181; 0.4 mW/mm^2^ vs 0.1 mW/mm^2^, p=0.0124; 0.2 mW/mm^2^ vs 0.1 mW/mm^2^, p=0.966). (**F, G**) Light-evoked Ca^2+^ increased with SpiRh1 (**F**) and SpiRh1[S186F] (**G**) in hindbrain V2a neurons. 3-dpf *Tg(vsx2:GAL4FF);Tg(UAS-hsp70l:SpiRh1-Flag-P2A-TagCFP, myl7:mCherry);Tg(UAS-hsp70l:GCaMP6s*) and *Tg(vsx2:GAL4FF);Tg(UAS-hsp70l:SpiRh1[S186F]-Flag-P2A-TagCFP, myl7:mCherry);Tg(UAS-hsp70l:GCaMP6s*) larvae were used. Sibling larvae that expressed GCaMP6s but did not express SpiRh1 or SpiRh1[S186F] were used as controls. The hindbrain area was irradiated and GCaMP6s fluorescence was detected with a fluorescence detection filter (excitation 470–495 nm, emission 510–550 nm) for SpiRh1. For SpiRh1[S186F], GCaMP6s fluorescence was detected after 1 s of 405 nm light application and filter conversion (about 4 s, shown in gray shade). Two larvae for each condition (SpiRh1, SpiRh1[S186F], and controls) were analyzed and three consecutive trials were analyzed. The change in fluorescence intensity of GCaMP6s (ΔF/F) is indicated as a ratio to the fluorescence intensity at the start of stimulation (**F**) for SpiRh1 and before (**G**) the start of stimulation with 405 nm light for SpiRh1[S186F]. The ΔF/F of Tg larvae expressing SpiRh1 or SpiRh1[S186F] is indicated by green lines whereas that of control larvae is indicated by black lines. Data from the three light applications are shown. Ca^2+^ responses were significantly higher in Tg larvae expressing SpiRh1 and SpiRh1[S186F] than control larvae. Linear mixed-effects model, * p<0.05, ** p<0.01, *** p<0.001, ns, not significant. Means and SEMs are shown. Figure 3—source data 1.Data for Figure 3, locomotion inducced by SpiRh1, SpiRh1[S186F], and ChRWR.

To evaluate the optogenetic activities of the G-protein-coupled rhodopsins in vivo, we expressed them in either hindbrain reticulospinal V2a neurons that were reported to drive locomotion (Kimura et al., 2013) or in cardiomyocytes of zebrafish larvae by using the Gal4-UAS system. Transgenic (Tg) zebrafish *Tg(vsx2:GAL4FF*), which is also known as *Tg(chx10:GAL4*), express a modified version of the transcriptional activator GAL4-VP16 (Asakawa et al., 2008) in hindbrain reticulospinal V2a neurons (Kimura et al., 2013). *Tg(myl7:GAL4FF*), in which GAL4FF was expressed under the control of the promoter of the cardiac myosin light chain gene *myl7* (Huang et al., 2003), was used to express rhodopsins in cardiomyocytes. We generated stable transgenic lines *Tg(UAS:opto-tool*) that can express Flag-tagged rhodopsins with P2A-TagCFP under the control of 5xUAS (upstream activating sequences of the yeast *Gal1* gene) and the zebrafish *hsp70l* promoter (Muto et al., 2017), and mCherry in the heart. We used an EGFP fusion protein of the channelrhodopsin wide receiver (ChRWR), which is a chimeric protein of *Chlamydomonas reinhardtii* channelrhodopsins ChR1 and ChR2, as a positive control (Kimura et al., 2013; Umeda et al., 2013; Wang et al., 2009). First, we crossed *Tg(vsx2:GAL4FF);Tg(UAS:RFP*) and *Tg(UAS:opto-tool*) to express various G-protein-coupled rhodopsins and ChRWR-EGFP, listed in Table 1, in hindbrain reticulospinal V2a neurons. The expression of these rhodopsins was examined by TagCFP or fused EGFP expression in 3 days post fertilization (3-dpf) Tg larvae, and was further analyzed by immunohistochemistry (Figure 2B, Table 1). Since transgene-mediated protein expression depends on the nature of the introduced gene, the transgene-integrated sites and copy number, we established multiple Tg lines and analyzed stable Tg lines (F_1_ or later generations) that expressed equally high - but varying - levels of these tools. We irradiated a hindbrain area of 3-dpf Tg larvae expressing the G-protein-coupled rhodopsins with light of wavelength near their absorption maxima to stimulate each rhodopsin, using a patterned illuminator (Figure 2A, Table 1). Six consecutive stimulus trials were analyzed for 8–12 rhodopsin-expressing or non-expressing sibling control larvae. Tail movements after light stimuli were monitored (Figure 2C–E, Figure 2—videos 1–3). The rate at which light application was able to induce tail movements (locomotion rate, Figure 3A), the time from irradiation to the onset of tail movements (latency, Figure 3B), the duration of tail movements (Figure 3C), and the amplitude of tail movements (strength, Figure 3D), were measured.

**Figure 2—video 1.** Tail movements in a larva expressing ChRWR-EGFP in reticulospinal V2a neurons. The hindbrain in a 3-dpf *Tg(vsx2:GAL4FF);Tg(UAS-hsp70l:ChRWR-EGFP);Tg(UAS:RFP*) larva was stimulated with 470 nm light for 100 ms. The timing of light stimulation is indicated by a blue circle.

**Figure 2—video 2.** Tail movements in a larva expressing SpiRh1 in reticulospinal V2a neurons. The hindbrain in a 3-dpf *Tg(vsx2:GAL4FF);Tg(UAS-hsp70l:SpiRh1-Flag-P2A-TagCFP, myl7:mCherry);Tg(UAS:RFP*) larva was stimulated with 520 nm light for 1 s. The timing of light stimulation is indicated by a green circle.

**Figure 2—video 3.** Tail movements in a larva expressing SpiRh1[S186F] in reticulospinal V2a neurons. The hindbrain in a 3-dpf *Tg(vsx2:GAL4FF);Tg(UAS-hsp70l:SpiRh1[S186F]-Flag-P2A-TagCFP; myl7:mCherry);Tg(UAS:RFP*) larva was stimulated with 405 nm light for 1 s. The timing of light stimulation is indicated by a purple circle.

**Figure 3—figure supplement 1.**
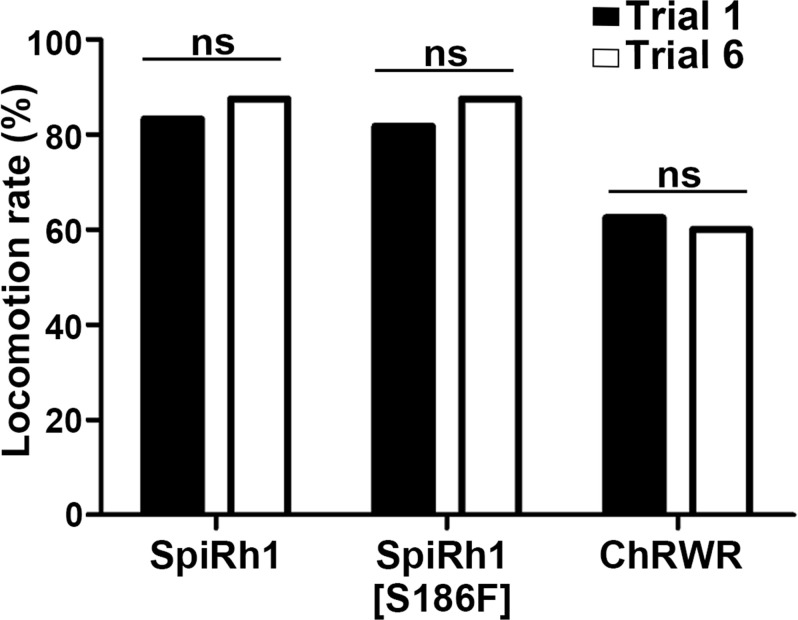
Effect of trial number on locomotion rate. Locomotion rates in trials 1 and 6 are shown (n=12 for SpiRh1 and SpiRh1[S186F], n=8 for ChRWR). Means and SEMs are indicated. ns, not significant; one-way ANOVA with Tukey’s post hoc test. Figure 3—figure supplement 1—source data 1.Data for Figure 3—figure supplement 1, effects of trial number on locomotion rate.

**Figure 3—figure supplement 2.**
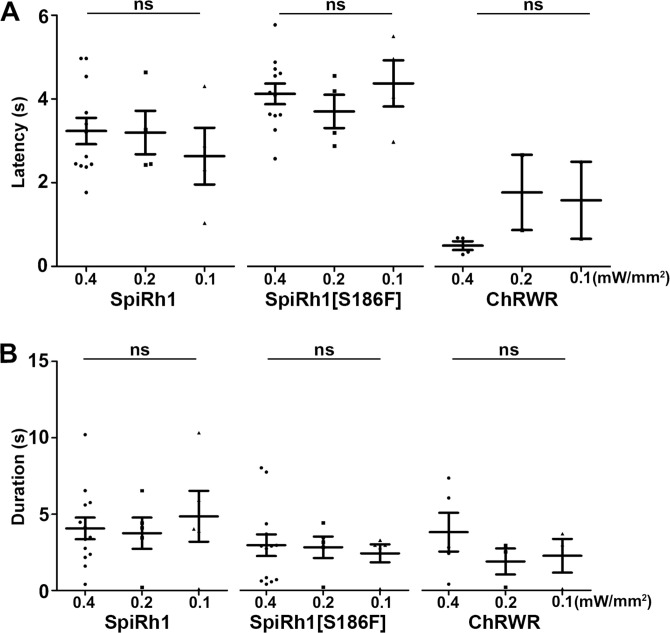
Latency and duration of locomotion induced with light of various intensities. Latency (**A**) and duration (**B**) of locomotion evoked with light of various intensities. The hindbrain region of 3-dpf larvae expressing SpiRh1, SpiRh1[S186F], and ChRWR in hindbrain reticulospinal V2a neurons was stimulated with light of various intensities (0.4, 0.2, or 0.1 mW/mm^2^). Six consecutive stimulation trials were analyzed for 4 or 12 larvae of each Tg line (n=12 for SpiRh1 and SpiRh1[S186F], n=4 for ChRWR). One-way ANOVA with Tukey’s post hoc test was used to statistically analyze data. ns: not significant. Means and SEMs are indicated. Figure 3—figure supplement 2—source data 1.Data for Figure 3—figure supplement 2, latency and duration of locomotion.

**Figure 3—video 1.** Ca^2+^ imaging of hindbrain reticulospinal V2a neurons of a larva expressing SpiRh1 and GCaMP6s. The hindbrain in 3-dpf *Tg(vsx2:GAL4FF);Tg(UAS-hsp70l:SpiRh1-Flag-P2A-TagCFP, myl7:mCherry);Tg(UAS-hsp70l:GCaMP6s*) was stimulated with a fluorescence detection filter (excitation 470–495 nm, emission 510–550 nm). The timing of stimulation is indicated by a blue square. GCaMP6s fluorescence was simultaneously monitored with the same filter.

**Figure 3—video 2.** Ca^2+^ imaging in hindbrain reticulospinal V2a neurons of a larva expressing SpiRh1[S186F] and GCaMP6s. The hindbrain in 3-dpf *Tg(vsx2:GAL4FF);Tg(UAS-hsp70l:SpiRh1[S186F]-Flag-P2A-TagCFP, myl7:mCherry);Tg(UAS-hsp70l:GCaMP6s*) was stimulated with 405 nm light after detection of the baseline fluorescence. The timing of light stimulation is indicated by a purple circle. GCaMP6s fluorescence was monitored with a fluorescence detection filter (excitation 470–495 nm, emission 510–550 nm). The timings of the observations are indicated by blue squares.

**Table 1.** Summary of optogenetic tools. Rhodopsins were expressed in hindbrain reticulospinal V2a neurons or cardiomyocytes using the Gal-4-UAS system. The wavelengths of light used in this study and the light for maximum activation or inhibition are described. The expression levels of the tools were determined by immunostaining with anti-Flag or anti-GFP (for ChRWR-EGFP) antibodies (+weak, ++medium, +++strong expression). The light stimulus-dependent responses are indicated by the percentage of fish that responded (induced swimming or cardiac arrest). As controls, the responses of sibling larvae that did not express the tool were also examined. The number of larvae analyzed are also indicated. *1 Light stimulus-dependent increase of cytoplasmic Ca^2+^. *2 Neither cardiac arrest, bradycardia, nor tachycardia were induced with either 490–510 nm, 530–560 nm (microscope-equipped light source, n=100), or 520 nm (LED, n=2) light. *3 Expression was confirmed by detecting TagCFP. *4 Cardiac arrest was not induced with 490–510 nm, 530–560 nm (microscope-equipped light source, n=60), or 620 nm (LED, n=2) light. *5 Light-stimulus-dependent reduction of cAMP. *6 The percentages of spontaneous tail movements elicited by white light that was inhibited by rhodopsin activation (locomotion-inhibition trials) are indicated (no rhodopsin activation was used as control). *7 Expression of ZPP1 and ZPP2 is shown in Figure 4—figure supplement 4. *8 Cardiac arrest was induced with 405 nm (LED) light for 1 s (n=2), and cardiac arrest was also induced with 426–446 nm (microscope-equipped light source, n*=*60) only when the interval between stimuli was more than 2–3 hr. *9 Cardiac arrest was not induced with 460–500 nm light (equipped with microscope). Abbreviations: NA, not available; ND, not determined; PP, parapinopsin; TMT, teleost multiple tissue (TMT) opsin.

GPCR type	Rhodopsin name	Origin	Light for activation[light for maximum activation](nm)	Light for inhibition[light for maximum inhibition](nm)	Cell response	V2a neurons		Heart	
						Expression	Response (control)	Expression	Response(control)
	ChRWR	*Chlamydomonas reinhardtii*	470 [470]	ND	ND	+++	73.8%, n=8 (8.75%, n=8)	ND	ND
Gq	Spider (Spi) Rh1	Jumping spider (*Hasarius adansoni*)	520 [520]	ND [NA]	++^*1^	++	89.0%, n=12 (15.0%, *n=8*)	+++	0%, n=102^*2^ (ND)
Gq	Spider (Spi) Rh1 [S186F]	Jumping spider (*Hasarius adansoni*)	405 [405]	ND [550]	++^*1^	+++	91.4%, n=12 (35.0%, n=8)	ND	ND
Gq	BeeUVOP	Honeybee (*Apis cerena*)	405 [405]	ND [>480]	ND	+^*3^	28.1%, n=8 (8.30%, n=8)	ND	ND
Gq	BeeBLOP	Honeybee (*Apis cerena*)	405 [405]	ND [>520]	ND	++	25.2%, n=8 (14.6%, n=8)	ND	ND
Gq	PxRh3	Butterfly (*Papilio xuthus*)	620 [620]	ND [520]	ND	+ +	35.0%, n=8 (20.0%, n=8)	ND	0%, n=62^*4^ (ND)
Gi/o	MosOpn3	Mosquito (*Anopheles stephensi*)	520 [520]	470–495 [470]	++^*5^	+^*3^	44.4%, n=3^*6^ (50.0%, n=3)	+++	100%, n=4 (0%, n=4)
Gi/o	PufTMT	Pufferfish (*Takifugu rubripes*)	470 [470]	470–495 [NA]	++^*5^	ND	ND	+++	100%, n=4 (0%, n=4)
Gi/o	LamPP	Lamprey (*Lethenteron camtschaticum*)	405 [405]	470–495 [520]	++^*5^	+^*3^	26.7%, n=7^*6^ (25.0%, n=7)	+++	100%, n=4 (0%, n=4)
Gi/o	ZPP1	Zebrafish (*Danio rerio*)	405 [405]	ND [520]	++^*5^	ND	ND	+++^*7^	100%, n=62^*8^ (ND)
Gi/o	ZPP2	Zebrafish (*Danio rerio*)	460–500 [470]	ND [NA]	ND	ND	ND	+^*7^	0%, n=100^*9^ (ND)

Among the G-protein-coupled rhodopsins examined, we found that SpiRh1 and SpiRh1[S186F] were most potent in inducing tail movements. Immunohistochemistry with anti-Flag or GFP antibodies revealed ChRWR expression on the cell surface of hindbrain reticulospinal V2a neurons was mosaic due to methylation-dependent silencing of the UAS system (Akitake et al., 2011), while SpiRh1 and SpiRh1[S186F] were uniformly expressed in these neurons (Figure 2B). As was previously reported (Kimura et al., 2013), light stimulation (0.4 mW/mm^2^) of reticulospinal V2a neurons with ChRWR for 100 ms immediately evoked tail movements (locomotion rate 73.8 ± 9.48%, latency 0.0555±0.00879 s) (Figures 2E, 3A and B, Figure 2—video 1). Activation with SpiRh1 and SpiRh1[S186F] required longer stimulation (1 s) and 3–5 s to initiate tail movements (SpiRh1 locomotion rate 89.0 ± 3.53%, latency 3.23±0.315 s; SpiRh1[S186F], locomotion rate 91.4 ± 3.9%, latency 4.12±0.246 s, Figures 2C, D, 3A and B, Figure 2—videos 2 and 3). However, stimulation with SpiRh1 and SpiRh1[S186F] elicited tail movements for a significantly longer duration than ChRWR (SpiRh1 4.37±0.691, SpiRh1[S186F] 3.17±0.735 s, ChRWR 0.684±0.226 s, Figure 3C). Light stimulation of control sibling larvae that did not express the rhodopsins scarcely induced tail movements, although stimulation with 405 nm light induced locomotion at a low frequency (locomotion rate of control larvae for SpiRh1, SpiRh1[S186F], ChRWR were 15.0 ± 5.88%, 35.0 ± 10.9%, 8.75 ± 3.33%, respectively, Figure 3A). Light stimulation with SpiRh1, SpiRh1[S186F], and ChRWR induced tail movements similarly in trials 1 and 6 (Figure 3—figure supplement 1). To analyze the photosensitivity of these rhodopsins, we applied light of various intensities (0.4, 0.2, and 0.1 mW/mm^2^) for 1 s. The rate of locomotion induced by ChRWR decreased when light intensity was reduced, that is, at 0.2 and 0.1 mW/mm^2^, while that with SpiRh1 and SpiRh1[S186F] did not change significantly when light intensity was reduced to 0.1 mW/mm^2^ (Figure 3E). The latency and duration of tail movements induced with SpiRh1 and SpiRh1[S186F] did not vary with different light intensities (Figure 3—figure supplement 2). These data indicate that optical activation of reticulospinal V2a neurons with SpiRh1 and SpiRh1[S186F] is robust and long-lasting, although it requires longer stimulation and longer latency than channelrhodopsin. In G-protein-mediated signaling, it is generally accepted that Gq activates PLCβ and thereby generates IP3, which induces Ca^2+^ influx from the endoplasmic reticulum. To examine the level of intracellular Ca^2+^ level, we expressed SpiRh1 or SpiRh1[S186F] with GCaMP6s in hindbrain reticulospinal V2a neurons. We found that light stimulation with these Gq-coupled rhodopsins increased the intracellular Ca^2+^ level in these neurons (Figure 3F and G, Figure 3—videos 1 and 2).

### Optogenetic manipulation of zebrafish heart by Gi/o-coupled rhodopsins

**Figure 4.**
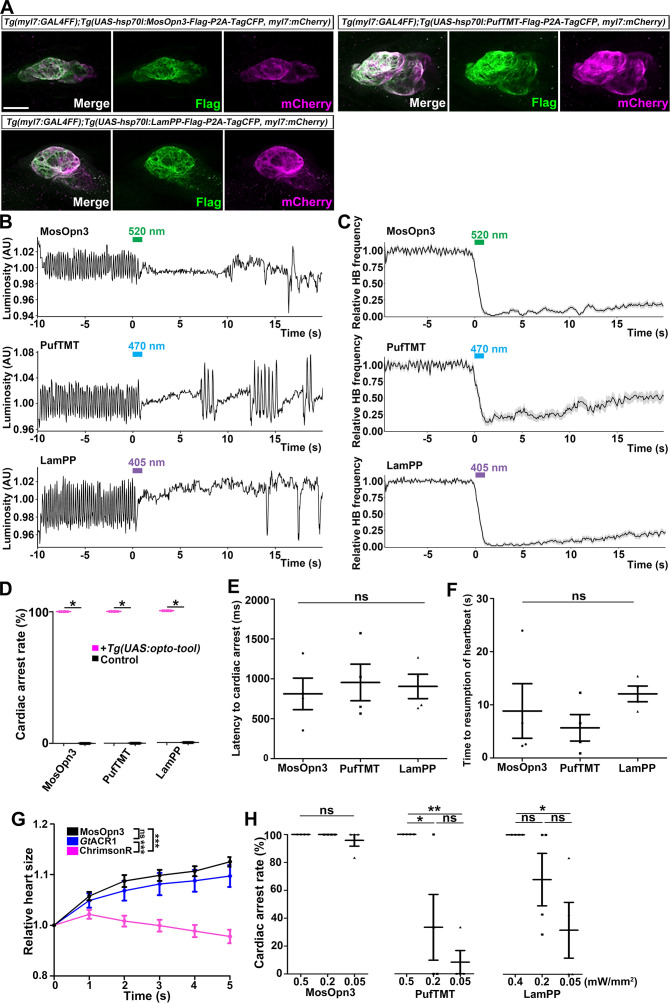
Inhibition of cardiomyocytes by Gi/o-coupled bistable rhodopsins. (**A**) Expression of Gi/o-coupled rhodopsins MosOpn3, PufTMT, and LamPP in zebrafish cardiomyocytes. 4-dpf *Tg(myl7:GAL4FF);Tg(UAS:opto-tool-Flag-P2A-TagCFP, myl7:mCherry*) larvae were fixed and stained with anti-Flag (green) and anti-DsRed (mCherry: magenta). (**B, C**) Heartbeat (HB) monitoring by change in luminosity (AU: arbitrary units) (**B**) and the average relative HB frequency (**C**) of four larvae expressing MosOpn3, PufTMT, and LamPP in cardiomyocytes. The heart area of larvae expressing MosOpn3, PufTMT, and LamPP in cardiomyocytes that were stimulated with 520, 470, and 405 nm light (0.5 mW/mm^2^), respectively, for 1 s. Six consecutive stimulus trials were analyzed for four rhodopsin-expressing larvae of each Tg line. The entire heart was manually set as the region of interest (ROI), and luminosity in the ROI was measured. The change in luminosity reflects the HB. The relative HB frequency was calculated from the HB data during 1 s before and after each time point. Six consecutive stimulus trials were analyzed for four rhodopsin-expressing larvae and four control larvae of each Tg line (MosOpn3, PufTMT, and LamPP). Typical HB data are shown in (**B**) and the average HB frequency for 24 trials are shown in (**C**). Gray shade indicates SEM. (**D**) Cardiac arrest rates. Wilcoxon rank sum test (MosOpn3, PufTMT, and LamPP, p=0.0131). (**E, F**) Latency to cardiac arrest (**E**), and time to resumption of HBs (**F**) with MosOpn3, PufTMT, and LamPP by light stimulation. One-way ANOVA followed by Tukey’s post hoc test was used for statistical analyses. (**G**) Heart size after activation of MosOpn3, *Gt*ACR1, and ChrimsonR in the heart. The heart area in the Tg larvae expressing MosOpn3, *Gt*ACR1-EYFP, or ChrimsonR-tdTomato was irradiated by a fluorescence detection filter (excitation 530–550 nm) for 5 s. The size of the entire heart area was measured, and the ratio to the size at the onset of cardiac arrest (t=0) was calculated and plotted in a graph. Five trials from two larvae for each condition were analyzed. The linear mixed effects model with Bonferroni-adjusted pairwise comparisons were used for statistical analyses. (**H**) Cardiac arrest rates induced by MosOpn3, PufTMT, and LamPP with light of various intensities. For MosOpn3 and PufTMT, one trial for 0.5 mW/mm^2^ and six consecutive trials for 0.2 or 0.05 W/mm^2^ were analyzed. For LamPP, one trial for 0.4 mW/mm^2^ and six consecutive trials for 0.2 and 0.05 W/mm^2^ were analyzed. One-way ANOVA with Tukey’s post hoc test (PufTMT 0.5 mW/mm^2^ vs 0.05 mW/mm^2^, p=0.00386; 0.5 mW/mm^2^ vs 0.2 mW/mm^2^, p=0.0239; LamPP 0.4 mW/mm^2^ vs 0.05 mW/mm^2^, p=0.0332). * p<0.05, ** p<0.01, *** p<0.001, ns: not significant. Means and SEMs are shown. Scale bar = 50 µm in (**A**). Figure 4—source data 1.Data for Figure 4, inhibition of cardiomyocytes by Gi/o-coupled rhodopsins.

Gi/o-coupled bistable rhodopsins MosOpn3 and LamPP were used to suppress neurotransmitter release (Copits et al., 2021; Mahn et al., 2021). We expressed Gi/o-coupled rhodopsin MosOpn3 and LamPP in hindbrain reticulospinal V2a neurons and examined whether they could suppress tail movements induced by a visual stimulus (white light). However, light stimulation of the hindbrain in zebrafish expressing MosOpn3 or LamPP did not suppress tail movements (Table 1). It is currently unknown why Gi/o-coupled rhodopsins did not suppress the activity of reticulospinal V2a neurons. Optogenetic control of cardiac function in zebrafish (Arrenberg et al., 2010) and mammals (Nussinovitch and Gepstein, 2015; Vogt et al., 2015; Watanabe et al., 2017) was reported previously. Thus, we examined whether Gi/o-coupled rhodopsins could be used to control cardiomyocyte function in vivo. By crossing *Tg(myl7:GAL4FF*) and *Tg(UAS:opto-tool*), we expressed Gi/o-coupled rhodopsins in cardiomyocytes. We again established multiple Tg lines and analyzed stable Tg lines that expressed equally high - but varying - levels of these tools. Immunohistochemical staining revealed comparable expression of these Gi/o-coupled rhodopsins in zebrafish cardiomyocytes (Figure 4A, Figure 4—figure supplement 4, Table 1). We irradiated the entire heart area of 4-dpf Tg larvae expressing the Gi/o-coupled rhodopsins with 0.5 mW/mm^2^ light of appropriate wavelengths (520 nm for MosOpn3, 470 nm for PufTMT, and 405 nm for LamPP) for 1 s. We applied light stimuli to each larva as six trials at intervals of 10 min and analyzed four Tg and control larvae for each type of rhodopsin. Videos of heartbeats (HBs) before and after light stimulation were recorded (Figure 4—video 1 for MosOpn3, Figure 4—video 2 for PufTMT, Figure 4—video 3 for LamPP). HBs were analyzed and heart rates were calculated (Figure 4B and C). Cardiac arrest rate, latency to cardiac arrest, and time to resumption of HB were determined (Figure 4D, E and F). Activation of the Gi/o-coupled rhodopsins MosOpn3, PufTMT, and LamPP in the heart led to cardiac arrest within approximately 1 s in all Tg larvae examined (812±198 ms for MosOpn3, 955±230 ms for PufTMT, and 905±153 ms for LamPP), but not in control sibling larvae (Figure 4B, C, D and E, Figure 4—videos 1–3). The first HB occurred about 10 s after cardiac arrest (8.83±5.13 s for MosOpn3, 5.67±2.49 s for PufTMT, and 12.1±1.48 s for LamPP, Figure 4F), but HBs gradually recovered and took at least 1 min (sometimes a few minutes) to return to normal (Figure 4B and C, Figure 4—videos 1–3). In trials 1 and 6, there was no significantly change in cardiac arrest following photoactivation by MosOp3 and LamPP, while a slight but significant difference was observed for PufTMT (Figure 4—figure supplement 1). These data suggest that MosOpn3, PufTMT, and LamPP are efficient optogenetic tools to control the function of cardiomyocytes in zebrafish.

**Figure 4—figure supplement 1.**
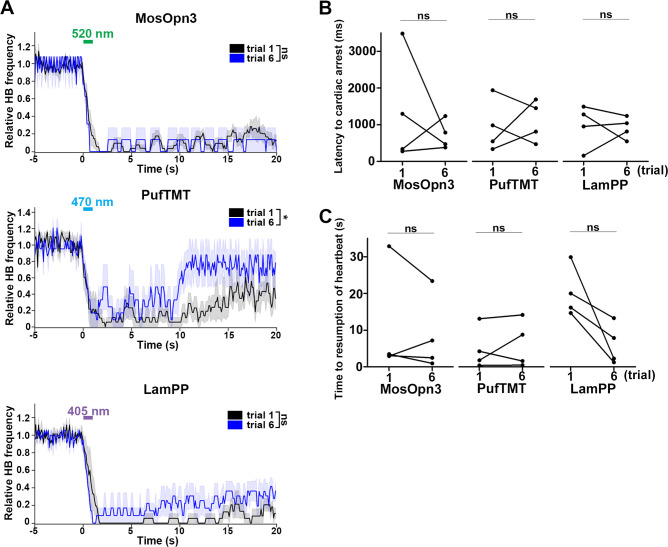
Effect of trial number on heartbeats (HBs). (**A**) Average relative HB frequency of larvae expressing MosOpn3 (n=7 for trial 1; n=2 for trial 6), PufTMT (n=5 for trial 1; n=3 for trial 6), and LamPP (n=4 for both trials 1 and 6) in cardiomyocytes. The heart area of Tg larvae was irradiated with light (520 nm for MosOpn3, 470 nm for PufTMT, and 405 nm for LamPP) at 0.5 mW/mm^2^ for 1 s at the indicated period. The statistical analysis employed the linear mixed effects model. Shading indicates SEM. (**B, C**) Latency to cardiac arrest (**B**) and time to resumption of HBs (**C**) in trials 1 and 6. Four larvae for each Tg line were analyzed. There were no significant differences in latency or time to resumption between trials 1 and 6 of MosOpn3, PufTMT, and LamPP. Student’s *t*-test, * p<0.05; ns, not significant. Figure 4—figure supplement 1—source data 1.Data for Figure 4—figure supplement 1, effect of trial number on HBs.

**Figure 4—figure supplement 2.**
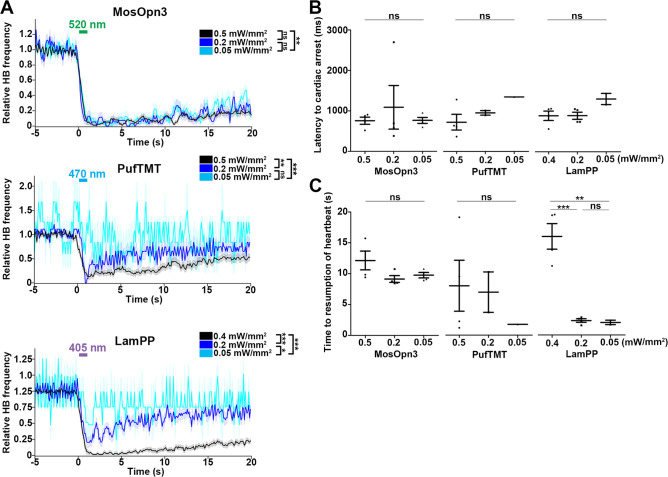
Change in heartbeats (HBs) after stimulation with light of various intensities. (**A**) Average relative HB frequency of larvae expressing MosOpn3, PufTMT, and LamPP in cardiomyocytes. The heart area of Tg larvae was irradiated with light (520 nm for MosOpn3, 470 nm for PufTMT, and 405 nm for LamPP) at 0.5, 0.2 or 0.05 mW/mm^2^ (for MosOpn3 and PufTMT), or 0.4, 0.2 or 0.05 mW/mm^2^ (for LamPP) for 1s at the indicated period. MosOpn3 (n=24 for 0.5 mW/mm^2^, n=18 for 0.2 mW/mm^2^, n=16 for 0.05 mW/mm^2^), PufTMT (n=24 for 0.5 mW/mm^2^, n=6 for 0.2 mW/mm^2^, n=4 for 0.05 mW/mm^2^), and LamPP (n=24 for 0.5 mW/mm^2^, n=18 for 0.2 mW/mm^2^, n=2 for 0.05 mW/mm^2^)-expressing larvae were analyzed. The statistical analysis employed the linear mixed effects model. Shading indicates SEM. (**B**) Latency to cardiac arrest (**B**) and time to resumption of HBs (**C**). Six consecutive trials were analyzed in four larvae for each condition. MosOpn3 (n=4 for 0.5, 0.2 and 0.05 mW/mm^2^), PufTMT (n=4 for 0.5 mW/mm^2^, n=2 for 0.2 mW/mm^2^, n=1 for 0.05 mW/mm^2^), and LamPP (n=4 for 0.4 mW/mm^2^, n=4 for 0.2 mW/mm^2^, n=2 for 0.05 mW/mm^2^)-expressing larvae were analyzed. One-way ANOVA with Tukey’s post hoc test (time to resumption of HBs LamPP 0.4 mW/mm^2^ vs 0.2 mW/mm^2^, p=0.000531; 0.2 mW/mm^2^ vs 0.05 mW/mm^2^, p=0.991; 0.4 mW/mm^2^ vs 0.05 mW/mm^2^, p=0.00158), * p<0.05. ** p<0.01. *** p<0.001; ns, not significant. Means and SEMs are indicated. Figure 4—figure supplement 2—source data 1.Data for Figure 4—figure supplement 2, change in HBs after stimulation of light of various intensities.

**Figure 4—figure supplement 3.**
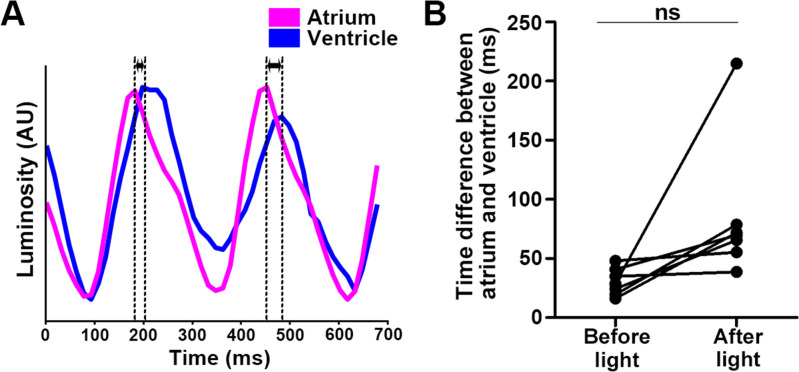
Time difference between atrial and ventricular contractions. (**A**) Heartbeats (HBs) of atrium and ventricle. The heart area of 4-dpf Tg larvae expressing MosOpn3 was irradiated with 520 nm light at an intensity of 0.5 mW/mm^2^ for 1 s. HB data were extracted from luminosity data of arbitrary positions of the atrium and ventricle. The time difference between atrial and ventricular (AV) contractions is indicated. (**B**) Time difference between AV contractions before and after light application. The 10 s period before light application is defined as ‘before light application’. The state in which the interval between HBs was twice as long as the interval before light application, but less than 5 s, is defined as ‘after light application’. Time differences between AV contractions before and after light applications were calculated and plotted in a graph. Seven trials were analyzed from six larvae. Student’s *t*-test; ns, not significant (p=0.0758). Figure 4—figure supplement 3—source data 1.Data for Figure 4—figure supplement 3, time difference between atrial and ventricular contractions.

**Figure 4—figure supplement 4.**
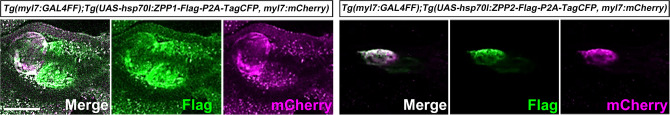
Expression of ZPP1 and ZPP2 in cardiomyocytes. Immunostaining of 4-dpf *Tg(myl7:GAL4FF);Tg(UAS-hsp70l:ZPP1-Flag-P2A-TagCFP, myl7:mCherry) Tg(myl7:GAL4FF); Tg(UAS-hsp70l:ZPP2-Flag-P2A-TagCFP, myl7:mCherry)* larvae, which were fixed and stained with anti-Flag (green) and anti-DsRed (mCherry: magenta) antibodies. Scale bar = 100 µm in (**A**).

**Figure 4—video 1.** Heartbeats in a larva expressing MosOpn3 in cardiomyocytes. The heart area of *Tg(myl7:GAL4FF);Tg(UAS-hsp70l:MosOpn3-Flag-P2A-TagCFP, myl7:mCherry)* was stimulated with 520 nm light for 1 s. The timing of light stimulation is indicated by a green circle.

**Figure 4—video 2.** Heartbeats in a larva expressing PufTMT in cardiomyocytes. The heart area of *Tg(myl7:GAL4FF);Tg(UAS-hsp70l:PufTMT-Flag-P2A-TagCFP, myl7:mCherry)* was stimulated with 470 nm light for 1 s. The timing of light stimulation is indicated by a blue circle.

**Figure 4—video 3.** Heartbeats in a larva expressing LamPP in cardiomyocytes. The heart area of *Tg(myl7:GAL4FF);Tg(UAS-hsp70l:LamPP-Flag-P2A-TagCFP, myl7:mCherry)* was stimulated with 405 nm light for 1 s. The timing of light stimulation is indicated by a purple circle.

**Figure 4—video 4.** Changes in heart size caused by activation of MosOpn3, *Gt*ACR1, and ChrimsonR in cardiomyocytes. Relaxation was caused by MosOpn3 and *Gt*ACR1, and contraction was caused by ChrimsonR. The heart area in Tg fish expressing MosOpn3, *Gt*ACR1, and ChrimsonR was irradiated with a fluorescence detection filter (excitation 530–550 nm, emission 575IF nm, U-MWIG3, Olympus) for 10 s.

To analyze how Gi/o-coupled rhodopsin induces cardiac arrest, we compared the effect of photoactivation of MosOpn3, anion channelrhodopsin *Gt*ACR1, and cation channelrhodopsin ChrimsonR on heart contraction (Figure 4G, Figure 4—video 4). *Gt*ACR1a and ChrimsonR can induce hyperpolarization and depolarization, respectively, in neurons (Antinucci et al., 2020; Govorunova et al., 2015; Klapoetke et al., 2014). Photoactivation of *Gt*ACR1 and ChrimsonR in cardiomyocytes for 5 s resulted in an increase and decrease, respectively of heart size. Photoactivation of MosOpn3 led to an increase in heart size, similar to *Gt*ACR1, suggesting that photoactivation of MosOpn3 suppresses heart contraction and induces cardiac arrest (Figure 4G, Figure 4—video 4). To analyze the photosensitivity of Gi/o-coupled rhodopsins, we applied light of various intensities for 1 s and examined the effect on HBs (Figure 4H, Figure 4—figure supplement 2). Cardiac arrest was induced and HB frequency remained low for over 20 s after 0.05 mW/mm^2^ light stimulation of MosOpn3 for 1 s. Photoactivation of PufTMT and LamPP at lower light intensities (0.2 or 0.05 mW/mm^2^) resulted in cardiac arrest at lower rates, but faster HB recovery than stimulation with 0.5 mW/mm^2^ light (Figure 4H, Figure 4—figure supplement 2). These data indicate that MosOpn3 more photosensitively suppressed HBs than PufTMT and LamPP in the zebrafish heart. We further examined atrial-ventricular (AV) conductivity by measuring the time difference between atrial and ventricular contractions before and after light stimulation when HBs had slightly recovered. There was no significant difference in AV conductivity before and after light stimulation (Figure 4—figure supplement 3).

Photoactivation of ZPP1 in the heart induced cardiac arrest for several seconds, while light stimulus-dependent cardiac arrest was not observed unless the time interval between stimuli exceeded 2–3 hr (Figure 4—figure supplement 4, Table 1). Photoactivation of SpiRh1 or SpiRh1[S186F] in cardiomyocytes did not induce cardiac arrythmia or arrest (Table 1).

### Switchable control of heartbeats by Gi/o-coupled rhodopsins

**Figure 5.**
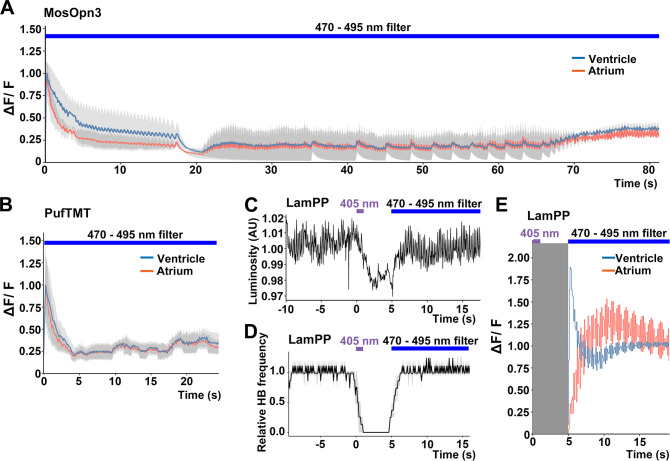
Switchable control of heartbeats by Gi/o-coupled bistable rhodopsins. (**A, B**) Average changes in fluorescence of GCaMP6s (ΔF/F) of 4-dpf larvae expressing MosOpn3 (**A**) or PufTMT (**B**), and GCaMP6s in cardiomyocytes. The heart area was irradiated with a fluorescence detection filter (excitation 470–495 nm, emission 510–550 nm) for the indicated period (n=2 for MosOpn3, n=4 for PufTMT). ΔF/F was calculated as a ratio to the fluorescence intensity of GCaMP6s at the start of stimulation. (**C, D**) HB monitoring by luminosity (AU) change (**C**) and average of relative HB frequency (n=2) (**D**) of 4-dpf larvae expressing LamPP in cardiomyocytes. The heart area was irradiated with 405 nm light (0.5 mW/mm^2^) for 1 s and then with a fluorescence detection filter (470–495 nm light) for the indicated period. Gray shading indicates SEMs (**A, B, D**). (**E**) Changes in ΔF/F of GCaMP6s of a larva expressing LamPP and GCaMP6s in the heart. The heart area was irradiated with 405 nm light (0.5 mW/mm^2^) for 1 s and then with a fluorescence detection filter (470–495 nm light) for the indicated period. GCaMP6s fluorescence was detected after light stimulation and filter conversion (5 s, shown in gray shading). ΔF/F was calculated as the ratio to the fluorescence intensity of GCaMP6s at the steady state (after the resumption of HBs). Blue and red lines indicate ΔF/F in the ventricle and atrium, respectively (**A, B, E**). Figure 5—source data 1.Data for Figure 5, switchable control of HBs by Gi/o-coupled rhodopsins.

Bistable rhodopsins convert to active states upon light stimulation, and then revert to the original inactive dark state by subsequent light absorption. Thus, the activity of these rhodopsins can be switched off by light stimulation after activation. The activation and inactivation wavelengths are close to each other for MosOpn3 and PufTMT, but apart for LamPP (Table 1). We assessed inactivation of the Gi/o-coupled rhodopsins by sustained light stimulation. We expressed MosOpn3, PufTMT, or LamPP together with GCaMP6s in cardiomyocytes, and simultaneously monitored intracellular Ca^2+^ and HBs. Continuous stimulation of MosOpn3 with 0.5 mW/mm^2^ light (470–495 nm) initially led to cardiac arrest and a reduction in intracellular Ca^2+^ concentration in both the atrium and ventricle of the heart within 20 s. However, HBs resumed and intracellular Ca^2+^ gradually increased around 40 s during light stimulation, and the HBs returned to a steady state at around 70 s (Figure 5A, Figure 5—video 1). Continuous light stimulation (0.5 mW/mm^2^, 470–495 nm) of PufTMT in the heart caused cardiac arrest and a reduction in intracellular Ca^2+^ concentration within about 5 s, followed by resumption of HBs in 5–10 s, and the return to a steady state at around 20 s (Figure 5B). These data suggest that sustained light stimulation can activate and subsequently inactivate MosOpn3 and PufTMT due to light adaptation. Stimulation of LamPP with 405 nm light in the heart led to cardiac arrest and a reduction in Ca^2+^, while subsequent sustained stimulation with 470–495 nm light recovered both heart rate and Ca^2+^ concentration (Figure 5C, D and E, Figure 5—videos 2 and 3). Therefore, the activity of LamPP can be turned on and off by using light of different wavelengths in the zebrafish heart.

**Figure 5—figure supplement 1.**
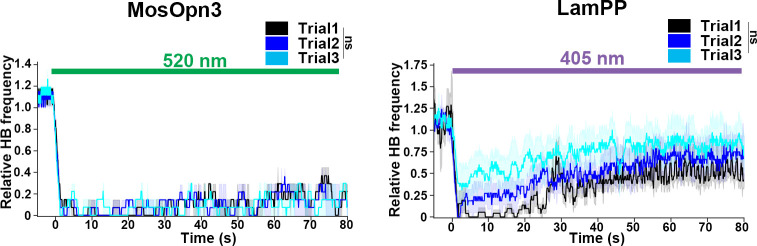
Average relative heartbeat frequency during prolonged irradiation. The heart area of Tg larvae expressing MosOpn3 or LamPP was irradiated for 80 s with 0.5 mW/mm^2^ light of 520 nm for MosOpn3 and 0.4 mW/mm^2^ light of 405 nm for LamPP. Three stimulation trials were performed with a 20 min interval in between. Two MosOpn3- and four LamPP-expressing larvae were analyzed. Linear mixed effects model; ns, not significant. Figure 5—figure supplement 1—source data 1.Data for Figure 5—figure supplement 1, average relative heartbeat frequency during prolonged irradiation.

**Figure 5—video 1.** Ca^2+^ imaging in the heart of a larva expressing MosOpn3 and GCaMP6s. The heart area of *Tg(myl7:GAL4FF);Tg(UAS-hsp70l:MosOpn3-Flag-P2A-TagCFP, myl7:mCherry);Tg(UAS-hsp70l:GCaMP6s)* was stimulated with a fluorescence detection filter (excitation 470–495 nm, emission 510–550 nm). GCaMP6s fluorescence was monitored with the same filter set. GCaMP6s fluorescence gradually decreased and became almost undetectable. At about 40 s, fluorescence recovered as the heart began to beat. A typical example is shown.

**Figure 5—video 2.** Changes in heartbeat (HB) following stimulation with light of different wavelengths in a larva expressing LamPP in cardiomyocytes. The heart area of *Tg(myl7:GAL4FF);Tg(UAS-hsp70l:LamPP-Flag-P2A-TagCFP, myl7:mCherry)* was stimulated with 405 nm light and a fluorescence detection filter (470–495 nm light). The timing of stimulation with 405 nm (1 s) and 470–495 nm light is indicated by a purple circle and a blue square, respectively. Cardiac arrest was induced by stimulation. Immediately after stimulation with 470–495 nm light, HBs were resumed. A typical example is shown.

**Figure 5—video 3.** Changes in heartbeat (HB) following light stimulation of LamPP in cardiomyocytes. Higher magnification view. The timing of stimulation with 405 nm (1 s) and 470–495 nm light (about 2.5 s) is indicated by a purple circle and a blue square, respectively. Cardiac arrest was induced by stimulation. Immediately after stimulation with 470–495 nm light, HBs were resumed. A typical example is shown.

Furthermore, we analyzed the light adaptation of Gi/o-coupled rhodopsins by repeating prolonged stimulation with light of a wavelength that only activates bistable rhodopsin. The hearts of Tg larvae expressing MosOpn3 or LamPP were irradiated with 0.5 mW/mm^2^ light of 520 nm for MosOpn3 or 0.4 mW/mm^2^ light of 405 nm for LamPP for 80 s in all three trials at 20 min intervals. During the photoactivation of MosOpn3, HBs recovered slightly after about 40 s in all trials. In contrast, HBs gradually recovered during the photoactivation of LamPP (Figure 5—figure supplement 1). Thus, during prolonged light stimulation, MosOpn3 maintained its active state for a relatively long period while LamPP transitioned to an inactive state more rapidly.

### Gi/o-coupled rhodopsins suppress the heart’s function through GIRKs

**Figure 6.**
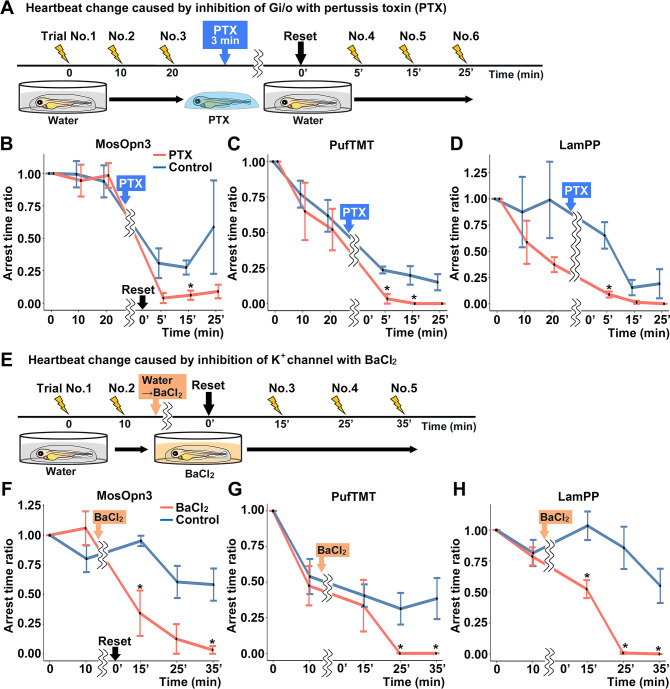
Gi/o and inward-rectifier K^+^ channel-dependent cardiac arrest by Gi/o-coupled bistable rhodopsins. (**A**) Time course of light application and treatment with pertussis toxin (PTX) (min, minutes). 4-dpf Tg larvae expressing MosOpn3, PufTMT, or LamPP in cardiomyocytes were used. After three trials of light stimulation of the heart area in larvae embedded in agarose, the larvae were treated with PTX for 3 min and embedded in agarose again and subjected to three subsequent light stimulation trials. In each trial, the heart area was irradiated with light (520 nm for MosOpn3, 470 nm for PufTMT, and 405 nm for LamPP) at an intensity of 0.5 mW/mm^2^ for 1 s, and cardiac arrest time was measured. The ratio to cardiac arrest time during the first trial was calculated (arrest time ratio). (**B, C, D**) Effect of PTX treatment on cardiac arrest induced by MosOpn3 (**B**), PufTMT (**C**), and LamPP (**D**). Average arrest time ratio of larvae expressing MosOpn3 (**B**), PufTMT (**C**), or LamPP (**D**) is shown in graphs. Larvae that were not treated with PTX were used as controls. Four treated and four non-treated control larvae were analyzed for each opto-tool. Wilcoxon rank sum test (MosOpn3 PTX vs control at 15 min, p=0.0294; PufTMT PTX vs control at 5 and 15 min, p=0.0265 and 0.0210; LamPP PTX vs control at 5 min, p=0.0285). (**E**) Time course of light application and treatment with BaCl_2_. After two trials of light stimulation of the heart area in larvae embedded in agarose, the larvae were treated with BaCl_2_ (or water) and subjected to three subsequent light stimulation trials. In each trial, the heart area was irradiated with light at an intensity of 0.5 mW/mm^2^ for 1 s. Cardiac arrest time was measured and the arrest time ratio was calculated. (**F, G, H**) Effect of BaCl_2_ treatment on cardiac arrest induced by MosOpn3 (**F**), PufTMT (**G**), and LamPP (**H**). Average arrest time ratio of larvae expressing MosOpn3 (**F**), PufTMT (**G**), or LamPP (**H**) is shown in graphs. Larvae that were not treated with BaCl_2_ were used as controls. Four treated and four non-treated control larvae were analyzed for each opto-tool. Wilcoxon rank sum test (MosOpn3 BaCl_2_ vs control at 15 and 35 min, p=0.0285 and 0.0265; PufTMT BaCl_2_ vs control at 25 and 35 min, p=0.0210 and 0.0210; LamPP BaCl_2_ vs control at 15, 25, and 35 min, p=0.0285, 0.0265, and 0.0210). * p<0.05. Means and SEMs are shown. Figure 6—source data 1.Data for Figure 6, GIRK-dependent cardiac arrest by Gi/o-coupled bistable rhodopsins.

To examine whether the optogenetic activity of MosOpn3, PufTMT, and LamPP depends on the activation of a Gi/o-type G protein, we treated the Tg fish expressing these rhodopsins with pertussis toxin (PTX), which induces ADP-ribosylation of Gαi and inhibits Gαi activity. For each Tg line, four PTX-treated and four non-treated control larvae were analyzed. We compared cardiac arrest time between PTX-treated fish and non-PTX-treated fish. Light-dependent activation of MosOpn3, PufTMT, or LamPP induced cardiac arrest. Cardiac arrest of these Gi/o-coupled rhodopsins was significantly suppressed by PTX treatment (Figure 6A, B, C and D, Figure 6—video 1), suggesting that optogenetic activity of these Gi/o-coupled rhodopsins requires the activation of the Gαi/o subunit.

**Figure 6—video 1.** Effect of pertussis toxin (PTX) treatment on cardiac arrest induced by PufTMT activation. 4-dpf Tg larvae expressing PufTMT in cardiomyocytes were stimulated with 470 nm light for 1 s in the first three trials. They were then treated with PTX (right panel) or remained untreated (left panel). Subsequently, they were stimulated with 470 nm light in trials 4–6. Heartbeats in trials 1 and 4 are shown. The timing of stimulation is indicated by blue circles. Cardiac arrest was no longer observed after PTX treatment (PTX Tx; trial 4, right panel).

**Figure 6—video 2.** Effect on BaCl_2_ treatment on cardiac arrest induced by LamPP activation. 4-dpf Tg larvae expressing LamPP in cardiomyocytes were stimulated with 405 nm light in trials 1 and 2. They were then treated with BaCl_2_ (right panel) or remained untreated (left panel). Subsequently, they were stimulated with 405 nm light in trials 3–5. Heart movements in trials 1 and 5 are shown. The timing of stimulation is indicated by purple circles. Cardiac arrest was no longer observed after BaCl_2_ treatment (BaCl_2_ Tx; trial 5, right panel).

Gi/o-coupled GPCRs are known to suppress adenylyl cyclase (AC) and reduce intracellular cAMP. They are also known to hyperpolarize cells by increasing K^+^ efflux through GIRKs (Hilger et al., 2018; Pierce et al., 2002; Rockman et al., 2002; Rosenbaum et al., 2009). To distinguish these two mechanisms, we treated Tg fish with BaCl_2_, an inhibitor of GIRKs, and compared cardiac arrest time between incubation with BaCl_2_ and water (control). For each Tg line, four BaCl_2_-treated and four non-treated control larvae were analyzed for each Tg line. The light stimulus-dependent cardiac arrest by MosOpn3, PufTMT, and LamPP was suppressed by incubation with BaCl_2_ (Figure 6E, F, G and H, Figure 6—video 2). The data suggest that the optogenetic activity of these Gi/o-coupled rhodopsins in the heart is dependent on GIRKs.

## Discussion

### Availability of animal bistable rhodopsins

We examined the optogenetic activities of G-protein-coupled bistable rhodopsins derived from various vertebrate and invertebrate animals in zebrafish neurons and cardiomyocytes. We found that Gq-coupled SpiRh1 and its derivative SpiRh[S186F] could activate Gq-mediated signaling in reticulospinal V2a neurons. Gi/o-coupled MosOpn3, PufTMT, and LamPP inhibited heart function when stimulated by light stimulation. Given that these bistable rhodopsins are sensitive to stimulating light of diverse wavelengths, they may be useful for manipulating various cell and tissue functions in vivo using light of different wavelengths. Animal bistable rhodopsins are endogenously expressed in various regions of the brain including photoreceptive tissues such as pineal and parapineal organs (Kawano-Yamashita et al., 2011; Kawano-Yamashita et al., 2020; Kawano-Yamashita et al., 2015; Kawano-Yamashita et al., 2007; Koyanagi et al., 2004; Koyanagi et al., 2015; Shen et al., 2021; Wada et al., 2012; Wada et al., 2021; Wada et al., 2018). If a wide area of the brain of Tg zebrafish is irradiated with white light, it may also activate endogenous bistable rhodopsins in addition to transgene-expressed rhodopsins and affect the functions of neurons or other tissues. It is, therefore, important to compare the effects of light stimulation between Tg and non-Tg control fish. In this study, patterned illumination of a specific area of the brain or heart with light of selected wavelength lights enabled us to control the functions of target cells in Tg but not in non-Tg fish (Figures 3A and 4D).

The bistable rhodopsins used in this study were photosensitive and functional without the addition of retinal derivatives in vivo. The bistable rhodopsins that bind to 11-*cis* retinal convert into an active state having all-*trans* retinal upon light absorption, and revert to the original inactive state by subsequent light absorption (Koyanagi et al., 2021; Koyanagi and Terakita, 2014; Terakita, 2005; Terakita et al., 2015). This bleach-resistant property confers activity to these bistable rhodopsins in non-photoreceptor cells. MosOpn3 was reported to bind to 13-*cis* retinal (Koyanagi et al., 2013). The 13-*cis* retinal-binding property of MosOpn3 assisted it to function in extraocular tissues since 13-*cis* retinal is generated in thermal equilibrium with the all-*trans* form, so 13-*cis* retinal is ubiquitously present (Terakita et al., 2015). In any case, our findings support that the bistable rhodopsins can be activated by light in various types of cells other than retinal cells.

### Light-dependent activation with Gq-coupled rhodopsins

We observed robust neuronal activation and an increase in Ca^2+^ in reticulospinal V2a neurons expressing Gq-coupled SpiRh1 and SpiRh1[S186F] (Figure 3). PLCβ mediates Gq-coupled signaling and produces IP_3_ and DAG from PIP_2_, which subsequently induces the release of Ca^2+^ from the ER and activates PKC and calmodulin kinases (CaMKs) (Hilger et al., 2018; Pierce et al., 2002; Rockman et al., 2002). It has been reported that binding of acetylcholine to a Gq-coupled muscarinic receptor (M1) activates non-selective cation channels and inhibits M-type K^+^ channels, inducing depolarization for a long period (Fisahn et al., 2002; Fraser and MacVicar, 1996; Hofmann and Frazier, 2010; McQuiston and Madison, 1999; Yue and Yaari, 2004). The inhibition of M-type K^+^ channels is considered to involve the PLCβ-mediated reduction of PIP_2_ (Brown, 2010). The same mechanism might be involved in neural activation, i.e. depolarization and generating action potentials, by SpiRh1 and SpiRh1[S186F]. It is also plausible that when Ca^2+^ increased, activated PKC and CaMKs phosphorylate cation channels, including neurotransmitter receptors, and this may also contribute to neural depolarization. This depolarization further leads to the activation of voltage-dependent calcium channels. Consistent with this event, a burst in Ca^2+^ was observed upon generation of action potentials after stimulation with SpiRh1 and SpiRh1[S186F] (Figure 3, Figure 3—videos 1 and 2). Although Gq-coupled PLCβ-mediated signaling takes more time than channelrhodopsin-mediated signaling to activate neurons, this feedforward mechanism likely contributes to robust and long-lasting neuronal activation.

Two types of rhodopsins, channelrhodopsin and Gq-coupled rhodopsins, were shown to activate reticulospinal V2a neurons (Figures 2 and 3; Kimura et al., 2013). Whereas photoactivation of channelrhodopsins immediately induced depolarization following cation influx, photoactivation of Gq-coupled rhodopsins induced a delayed increase in Ca^2+^ and neuronal activation. Similar neural activation takes place by binding of neurotransmitters to their receptors. For example, binding of glutamate to ion channel-type AMPA receptors and GPCR-type metabotropic receptors (mGluRs), which are often present on the same postsynaptic membrane, likely induces immediate depolarization and a delayed Ca^2+^/depolarization pathway. While the depolarization signal directly participates in the information transmission of neural circuits, the increase in intracellular Ca^2+^ may regulate changes in synaptic transmission efficiency by modifying neurotransmitter receptors and/or channels and controlling their function and localization. Given that the two signals have different roles in neural circuit function, SpiRh1 and SpiRh1[S186F], together with channelrhodopsins, may be helpful in distinguishing the roles of these two signals in neural circuit function.

Optogenetic activation of Gq-coupled neuropsin was shown to increase heart rate in mice (Wagdi et al., 2022). However, light stimulation with SpiRh1 in zebrafish cardiomyocytes did not apparently affect heart function (Table 1). It is unknown why activation of SpiRh1 in the heart did not lead to an increase in heart rate. The contraction of heart muscles and the control of heart rate requires an increase in intracellular Ca^2+^. It remains elusive whether SpiRh1 activation does not induce a sufficient increase in Ca^2+^ to affect heart function, or whether cooperation of action potentials together with an increase in Ca^2+^ is required for optic control of heart function in zebrafish. Future studies with calcium and voltage imaging and/or optogenetic activation of multiple pathways may clarify this issue.

### Optogenetic control of zebrafish heart with Gi/o-coupled bistable rhodopsins

Light stimulation of Gi/o-coupled rhodopsins MosOpn3, PufTMT, and LamPP in the heart-induced cardiac arrest (Figure 4). The effect of short-term activation of these Gi/o-coupled rhodopsins on cardiac function was persistent. The effect of trial number was minimal, although there were some differences among the rhodopsins (Figure 4—figure supplement 1). In addition, a dependence on stimulus light intensities was observed (Figure 4). Therefore, these Gi/o-coupled rhodospins are controllable and robust optogenetic tools for studying zebrafish cardiac function. Several physiological mechanisms could be considered for inducing cardiac arrest through the activation of Gi/o-coupled rhodopsins, including changes in myocardial contractility, conduction velocity in the AV node, and HB rhythm (pacemaker). In this study, rhodopsins were expressed in all cardiomyocytes, although detailed mechanisms have not been fully elucidated. Nonetheless, we carried out some additional experiments to offer greater clarity. First, the state of cardiac contraction induced by activation of MosOpn3 was compared to the states of a relaxed heart mediated by anion channelrhodopsin *Gt*ACR1 and a contracted heart mediated by cation channelrhodopsin ChrimsonR (Figure 4). Those results revealed that hearts experiencing cardiac arrest following activation of MosOpn3 were in a relaxed state. This is consistent with the concept that Gi/o-couple rhosopsins induce hyperpolarization of cardiomyocytes through GIRKs (see below).

MosOpn3 gradually restored normal HBs following light stimulus (Figure 4), indicating a transition to an inactive state during this process. AV conductivity was examined in two conditions: during the transition phase of MosOpn3’s partial activation (during recovery) and in the absence of MosOpn3 activation. However, no significant difference was observed between these two conditions. This suggests that conductivity in the AV node might not be affected. However, it is necessary to investigate the expression of MosOpn3 in the AV node and the effects of localized irradiation on the AV node in Tg fish. The influence on cardiac rhythm was not assessed in this study. Future studies using electrocardiograms and electrophysiological analyses using zebrafish Tg fish will clarify what aspects of heart functions can be controlled by Gi/o-coupled rhodopsins.

### Mechanisms of Gi/o-coupled rhodopsin-mediated heart control

The effect of Gi/o-coupled rhodopsins on cardiac arrest was inhibited by treatment with PTX and BaCl_2_ (Figure 6), suggesting that the Gi/o-coupled rhodopsins suppress neuronal activity by K^+^ channel-mediated hyperpolarization, which is mediated by the Gβγ subunit. It was previously reported that MosOpn3 and LamPP decreased neuronal excitability by coupling to GIRKs, but they also suppressed neurotransmitter release by inhibiting voltage-dependent Ca^2+^ channels at presynaptic terminals (Copits et al., 2021; Mahn et al., 2021). It is possible that the PTX and BaCl_2_ treatments might have affected the functional expression of endogenous Gi/o-coupled GPCRs and indirectly affected the activity of the Gi/o-coupled rhodopsins. However, considering the complete suppression of light-induced cardiac arrest (Figure 6), these Gi/o-coupled rhodopsins likely suppressed the heart’s function through GIRKs in cardiomyocytes. As Gi/o-coupled GPCRs also regulate intracellular cAMP level via AC regulation, light stimulation of MosOpn3, PufTMT, LamPP, or ZPP1 reduced cAMP levels in HEK293S cells (Figure 1). The Gi/o-mediated control of cell functions may depend on cell type and subcellular location. We expressed MosOpn3, PufTMT, and LamPP in reticulospinal V2a neurons, although light activation of these Gi-coupled rhodopsins did not suppress spontaneous tail movements (Table 1). The inability to suppress tail movements may be due to slow activation of Gi/Go-mediated signaling by these bistable rhodopsins or the lack of other components in V2a neurons. Optimization of these tools and stimulation methods may be necessary, depending on cell type.

### Bistable nature of G-coupled rhodopsins

A short duration of light stimulation (1 s) of the heart expressing MosOpn3 or PufTMT induced cardiac arrest, resumed HBs after 10 s, and returned to a steady state after a few minutes (Figure 4), while prolonged light application returned HBs to a steady state in a shorter time after cardiac arrest than short light application (Figure 5). As the wavelengths of light effective for activation and inactivation were close for MosOpn3 and PufTMT, light application likely induced both activation and inhibition of these Gi-coupled bistable rhodopsins. In contrast, the light wavelengths for activation and inactivation were apart for LamPP, which is switchable between these two states (Copits et al., 2021; Koyanagi et al., 2004; Rodgers et al., 2021). Consistent with this, cardiac arrest was induced by 405 nm light with LamPP, while irradiation of around 470 nm light resumed HBs (Figure 5C, D and E), suggesting that LamPP can be turned on and off by different wavelengths of light in the zebrafish heart. Like LamPP, ZPP1 has different light wavelengths for activation and inactivation (Table 1). However, photoactivation of ZPP1 resulted in only a short period of cardiac arrest and its photosensitivity did not recover for a few hours. The photoproduct (active form) of ZPP1 might not be stable (i.e. it might release the chromophore easily) compared to that of MosOpn3, PufTMT, and LamPP in zebrafish cardiomyocytes.

Since Gq-coupled SpiRh1, and Gi-coupled MosOpn3, PufTMT, and LamPP are bistable rhodopsins, their photoproducts, which activate G protein-mediated signaling, are considered to be stable unless they receive inactivating light. The tail movements stopped several seconds after stimulation with SpiRh1 and SpiRh1[S186F], and HBs resumed a few minutes after stimulation with MosOpn3, PufTMT, and LamPP (Figures 2—4), suggesting that activity of the bistable rhodopsins gradually reduced after transient stimulation. During prolonged stimulation with light, MosOpn3 maintained cardiac arrest for about 40 s, while LamPP exhibited gradual recovery of HB frequency (Figure 5—figure supplement 1). This observation suggests that MosOpn3 exhibits only slight light adaptation while LamPP is more susceptible to inactivation due to light adaptation. Therefore, despite differences among these bistable rhodopsins, there are likely intrinsic light adaptation mechanisms that inactivate bistable rhodopsins other than the photo-dependent reversal from an active to an inactive form. These mechanisms might not involve the release of all-*trans* retinal, but instead involve the phosphorylation-dependent binding of β-arrestin to rhodopsins and the β-arrestin-mediated internalization of rhodopsins (Kawano-Yamashita et al., 2011). In any case, by using G-protein-coupled bistable rhodopsins with different properties (activating/inactivating light wavelengths, stability, etc.), the functions of cells and tissues can be finely controlled by light stimulation.

### Utility of bistable rhodopsin to study cell and tissue functions

Optogenetic tools that are proven to be useful in mammals are also effective in zebrafish, and vice versa. The bistable rhodopsin tools that we designed are effective in zebrafish, but are also active in human HEK293S cells (Table 1). Bistable rhodopsins were shown to be expressed in mammalian tissues and used to optogenetically manipulate GPCR signaling in vivo (Copits et al., 2021; Dai et al., 2022; Mahn et al., 2021; Makowka et al., 2019; Rodgers et al., 2021; Wagdi et al., 2022). In this study, the expression plasmids for bistable rhodopsins were constructed to express tagged rhodopsin and P2A-TagCFP by the Gal4-UAS system in specific types of zebrafish cells. As small epitope-tagged bistable rhodopsins were more active than fluorescent protein-fused rhodopsins (Figure 1), they could also be more active in cells of other species, including mammals.

In this study, zebrafish larvae were used to study the role of GPCR signaling in cardiac function. Differences in heart structure and function were found between larvae and adult zebrafish. As a zebrafish grows, blood pressure increases and the heart becomes more complex, developing valves and ventricular trabeculae (Hu et al., 2000). Therefore, GPCR signaling, which regulates heart structure and function, may differ between juvenile and adult fish. Optogenetic manipulation of the heart’s function in adult zebrafish using bistable opsins should clarify this issue.

The genome of a single vertebrate species contains hundreds of GPCR genes. Many GPCRs function as receptors for sensations (e.g. odorant and taste receptors), and some function as receptors of some endogenous ligands (Pierce et al., 2002). There are also many GPCR signals whose role in vivo is not yet known. In the nervous system, GPCRs function as metabotropic receptors for neurotransmitters and neuromodulators, and are involved in neuronal functions such as synaptic plasticity, involving long-term potentiation (LTP) or depression (LTD) in neural circuits (Reiner and Levitz, 2018). Optogenetic manipulation of individual GPCR signaling should lead to a better understanding of their roles in synaptic plasticity and neural circuits. GPCRs also play important roles in regulating the function of internal organs (de Lucia et al., 2018; Pierce et al., 2002; Rockman et al., 2002). Certain GPCRs that share ligands are known to activate multiple signaling pathways and confer diverse cellular responses. They can interact with multiple types of G proteins. For example, there are three types of adrenergic receptors (ARs), α1, α2, and β, which bind to Gq, Gi/o, and Gs, respectively (β2 and β3 also bind to Gi), and activate different downstream signaling pathways (Hilger et al., 2018; Pierce et al., 2002; Rockman et al., 2002; Rosenbaum et al., 2009). Using optogenetic techniques, it may be possible to distinguish the in vivo roles of these adrenergic receptors and other GPCRs. The G-coupled bistable rhodopsins analyzed in this study may be useful tools for the optogenetic control of various cell and tissue functions.

## Materials and methods

**Key resources table:** 

Reagent type (species) or resource	Designation	Source or reference	Identifiers	Additional information
Gene (*Chlamydomonas reinhardtii*)	ChRWR-EGFP	Umeda et al., 2013		
Gene (*Hasarius adansoni)*	Spider Rh1 (SpiRh1)	Koyanagi et al., 2008; Nagata et al., 2012	GenBank: AB251846 Human codon optimized	
Gene (*Hasarius adansoni)*	Spider Rh1[S186F] (SpiRh1[S186F])	Nagata et al., 2019	GenBank: AB251846 S186F mutation is introduced	
Gene (*Apis cerana*)	Honeybee UV opsin (beeUVOP)	Terakita et al., 2008	Genbank: AB355816	
Gene (*Apis cerana*)	Honeybee blue opsin (beeBLOP)	Terakita et al., 2008	Genbank: AB355817	
Gene (*Papilio xuthus*)	Butterfly PxRh3	Saito et al., 2019	Genbank: AB007425	
Gene (*Anopheles stephensi*)	Mosquito Opn3 (MosOpn3)	Koyanagi et al., 2013	Genbank: AB753162 Carboxy terminal truncated	
Gene (*Takifugu rubripes*)	Pufferfish TMT opsin (PufTMT)	Koyanagi et al., 2013	Genbank: AF402774	
Gene (*Lethenteron camtschaticum*) parapinopsin	Lamprey (LamPP)	Koyanagi et al., 2004	Genbank: AB116380	
Gene (*Danio rerio*)	Zebrafish parapinopsin 1 (ZPP1)	Koyanagi et al., 2015	Genbank: AB626966	
Gene (*Danio rerio*)	Zebrafish parapinopsin 2 (ZPP2)	Koyanagi et al., 2015	Genbank: AB626967	
Gene (porcine teschovirus-1)	Porcine teschovirus 2 A (P2A)	Tanabe et al., 2010		
Genetic reagent (*Danio rerio*)	*mitfa^w2/w2^*	Lister et al., 1999	RRID:ZFIN_ZDB-GENO-070501-2	
Genetic reagent (*Danio rerio*)	*TgBAC(vsx2:GAL4FF)*	Kimura et al., 2013	*TgBAC(vsx2:GAL4FF) nns18Tg*	
Genetic reagent (*Danio rerio*)	*Tg(myl7:GAL4FF)*	This paper	*Tg(myl7:GAL4FF)nub38Tg*	Available from M. Hibi lab
Genetic reagent (*Danio rerio*)	*Tg(UAS:ChRWR-EGFP)*	Kimura et al., 2013	*Tg(UAS:ChRWR-EGFP)js3Tg*	
Genetic reagent (*Danio rerio*)	*Tg(UAS-hsp70l:SpiRh1-Flag-P2A-TagCFP)*	This paper	*Tg(5xUAS-hsp70l:Had.Rh1-Flag-P2A-TagCFP, myl7:mCherry)nub39Tg*	Available from M. Hibi lab
Genetic reagent (*Danio rerio*)	*Tg(UAS-hsp70l:SpiRh1[S186F]-Flag-P2A-TagCFP)*	This paper	*Tg(5xUAS-hsp70l:Had.Rh1[S186F]-Flag-P2A-TagCFP, myl7:mCherry)nub40Tg*	Available from M. Hibi lab
Genetic reagent (*Danio rerio*)	*Tg(UAS-hsp70l:beeUVOP-Flag-P2A-TagCFP)*	This paper	*Tg(5xUAS-hsp70l: Ace.UVOP-Flag-P2A-TagCFP, myl7:mCherry)nub41Tg*	Available from M. Hibi lab
Genetic reagent (*Danio rerio*)	*Tg(UAS-hsp70l:beeBLOP-Flag-P2A-TagCFP)*	This paper	*Tg(5xUAS-hsp70l:Ace.BLOP-Flag-P2A-TagCFP, myl7:mCherry)nub42Tg*	Available from M. Hibi lab
Genetic reagent (*Danio rerio*)	*Tg(UAS-hsp70l:PxRh3-Flag-P2A-TagCFP)*	This paper	*Tg(5xUAS-hsp70l:Pxu.Rh3-Flag-P2A-TagCFP; myl7:mCherry)nub43Tg*	Available from M. Hibi lab
Genetic reagent (*Danio rerio*)	*Tg(UAS-hsp70l:MosOpn3-Flag-P2A-TagCFP)*	This paper	*Tg(5xUAS-hsp70l:Ast.Opn3-Flag-P2A-TagCFP, myl7:mCherry)nub44Tg*	Available from M. Hibi lab
Genetic reagent (*Danio rerio*)	*Tg(UAS-hsp70l:PufTMT-Flag-P2A-TagCFP)*	This paper	*Tg(5xUAS-hsp70l:Tru.TMT-Flag-P2A-TagCFP, myl7:mCherry)nub45Tg*	Available from M. Hibi lab
Genetic reagent (*Danio rerio*)	*Tg(UAS-hsp70l:LamPP-Flag-P2A-TagCFP)*	This paper	*Tg(5xUAS-hsp70l:Lca.PP-Flag-P2A-TagCFP, myl7:mCherry)nub46Tg*	Available from M. Hibi lab
Genetic reagent (*Danio rerio*)	*Tg(UAS-hsp70l:ZPP1-Flag-P2A-TagCFP)*	This paper	*Tg(5xUAS-hsp70l:parapinopsina-Flag-P2A-TagCFP, myl7:mCherry)nub47Tg*	Available from M. Hibi lab
Genetic reagent (*Danio rerio*)	*Tg(UAS-hsp70l:ZPP2-Flag-P2A-TagCFP)*	This paper	*Tg(5xUAS-hsp70l:parapinopsinb-Flag-P2A-TagCFP, myl7:mCherry)nub48Tg*	Available from M. Hibi lab
Genetic reagent (*Danio rerio*)	*Tg(UAS-hsp70l:GCaMP6s)*	Muto et al., 2017	*Tg(5xUAS-hsp70l:GCaMP6s) nkUAShspzGCaMP6s13aTg*	
Genetic reagent (*Danio rerio*)	*Tg(UAS-hsp70l:GtACR1-EYFP)*	This paper	*Tg(5xUAS-hsp70l:GtACR1-EYFP, myl7:mCherry)nub53Tg*	Available from M. Hibi lab
Genetic reagent (*Danio rerio*)	*Tg(UAS-hsp70l:ChrimsonR-tdTomato)*	This paper	*Tg(5xUAS-hsp70l:ChrimsonR-tdTomato)nub119Tg*	Available from M. Hibi Lab
Cell line (*Homo sapiens*)	Human embryonic kidney 293 S (HEK293S)	Terakita et al., 2008		
Recombinant DNA reagent	pCS2 +SpiRh1-Flag-P2A-TagCFP	This paper		Mammalian expression plasmid, available from M. Hibi lab
Recombinant DNA reagent	pCS2 +SpiRh1[S186F]-Flag-P2A-TagCFP	This paper		Mammalian expression plasmid, available from M. Hibi lab
Recombinant DNA reagent	pCS2 +MosOpn3-Flag-P2A-TagCFP	This paper		Mammalian expression plasmid, available from M. Hibi lab
Recombinant DNA reagent	pCS2 +PufTMT-Flag-P2A-TagCFP	This paper		Mammalian expression plasmid, available from M. Hibi lab
Recombinant DNA reagent	pCS2 +LamPP-Flag-P2A-TagCFP	This paper		Mammalian expression plasmid, available from M. Hibi lab
Recombinant DNA reagent	pCS2 +ZPP1-Flag-P2A-TagCFP	This paper		Mammalian expression plasmid, available from M. Hibi lab
Recombinant DNA reagent	pGloSesor-20F cAMP	Promega	GeneBank: EU770615.1	
Recombinant DNA reagent	pcDNA3.1+/mit-2mutAEQ	Addgene #45539		
Recombinant DNA reagent	pT2ALR-Dest	Dohaku et al., 2019		Tol2 Gateway plasmid, available from M. Hibi lab
Recombinant DNA reagent	pBH-R1-R2	This paper		Tol2 Gateway Plasmid, available from M. Hibi lab
Recombinant DNA reagent	pENTR L1-5xUAS-hsp70l-R5	This paper		Gateway entry clone, available from M. Hibi lab
Recombinant DNA reagent	pENTR L5-SpiRh1-Flag-P2A-TagCFP-SV40pAS-L2	This paper		Gateway entry clone, available from M. Hibi lab
Recombinant DNA reagent	pENTR L5-SpiRh1[S186F] -Flag-P2A-TagCFP-SV40pAS -L2	This paper		Gateway entry clone, available from M. Hibi lab
Recombinant DNA reagent	pENTR L5-beeUVOP-Flag-P2A-TagCFP-SV40pAS-L2	This paper		Gateway entry clone, available from M. Hibi lab
Recombinant DNA reagent	pENTR L5-beeBlueOP-Flag-P2A-TagCFP-SV40pAS-L2	This paper		Gateway entry clone, available from M. Hibi lab
Recombinant DNA reagent	pENTR L5-PxRh3-Flag-P2A-TagCFP-SV40pAS-L2	This paper		Gateway entry clone, available from M. Hibi lab
Recombinant DNA reagent	pENTR L5-MosOpn3-Flag-P2A-TagCFP-SV40pAS-L2	This paper		Gateway entry clone, available from M. Hibi lab
Recombinant DNA reagent	pENTR L5-PufTMT-Flag-P2A-TagCFP-SV40pAS-L2	This paper		Gateway entry clone, available from M. Hibi lab
Recombinant DNA reagent	pENTR L5-LamPP-Flag-P2A-TagCFP-SV40pAS-L2	This paper		Gateway entry clone, available from M. Hibi lab
Recombinant DNA reagent	pENTR L5-ZPP1-Flag-P2A-TagCFP-SV40pAS-L2	This paper		Gateway entry clone, available from M. Hibi lab
Recombinant DNA reagent	pENTR L5-ZPP2-Flag-P2A-TagCFP-SV40pAS-L2	This paper		Gateway entry clone, available from M. Hibi lab
Antibody	Mouse monoclonal anti-Flag antibody	Sigma-Aldrich	Cat# F3165; RRID:AB_259529	Dilution 1:500
Antibody	Mouse monoclonal anti-Myc tag antibody	Santa Cruz Biotechnology	Cat# sc-40; RRID:AB_627268	Dilution 1:500
Antibody	Rat monoclonal anti-GFP antibody	Nacalai Tesque, Inc	Cat# 04404–84; RRID:AB_10013361	Dilution 1:500
Antibody	Rabbit polyclonal anti-DsRed antibody	Takara Bio	Cat# 632496; RRID:AB_10013483	Dilution 1:500
Antibody	Goat CF488A anti-mouse IgG antibody	Biotium Inc	Cat# 20018; RRID:AB_10557263	Dilution 1:500
Antibody	Goat CF488A anti-rat IgG antibody	Biotium, Inc	Cat# 20023; RRID:AB_10557403	Dilution 1:500
Antibody	Goat CF568 anti-rabbit IgG antibody	Biotium Inc	Cat# 20103; RRID:AB_10558012	Dilution 1:500
Chemical compound, drug	YM-254890	Fujifilm Wako Pure Chemical Corp.	257–00631	
Chemical compound, drug	low gelling temperature Type VII-A	Sigma-Aldrich	A0701	
Chemical compound, drug	tricaine methanesulfonate	Nacalai Tesque, Inc	Cat# 886-86-2	
Chemical compound, drug	pentylenetetrazol	Sigma-Aldrich	Cat# P6500	
Chemical compound, drug	Pertussis toxin	FUJIFILM Wako Pure Chemical Corp.	Cat# 168–22471	
Chemical compound, drug	BaCl_2_	FUJIFILM Wako Pure Chemical Corp.	Cat# 025–00172	
Software, algorithm	PolyScan2	Mightex		
Software, algorithm	StreamPix7	NorPix Inc		
Software, algorithm	LabVIEW	National Instruments	2015	https://www.ni.com/ja-jp.html
Software, algorithm	GraphPad Prism5	GraphPad Software		https://www.mdf-soft.com/
Software, algorithm	VSDC Free Video Editor 6.4.7.155	FLASH-INTEGRO LLC		https://www.videosoftdev.com/jp
Software, algorithm	Microsoft Movies & TV	Microsoft Corp.		https://apps.microsoft.com/store/detail/movies-tv/9WZDNCRFJ3P2
Software, algorithm	QuickTime player 10.5	Apple Inc		https://quicktime.softonic.jp/
Software, algorithm	Fiji / ImageJ	National Institutes of Health (NIH)		http://fiji.sc/
Software, algorithm	R 3.6.1 and 4.2.1			https://www.r-project.org/
Software, algorithm	ggplot2 3.2.0 of R			https://ggplot2.tidyverse.org/
Software, algorithm	nlme 3.1–162 of R			https://cran.r-project.org/web/packages/nlme/index.html
Software, algorithm	Bonsai	Lopes et al., 2015		https://open-ephys.org/bonsai
Software, algorithm	Python 3.5.6	Python Software Foundation		https://www.python.org/
Software, algorithm	Tracker Video Analysis and Modeling Tool for Physics Education 5.1.5			https://physlets.org/tracker/
Software, algorithm	Microsoft Excel for Mac, ver. 16.74	Microsoft		
Software, algorithm	HB_frequency.py	This paper		Source code file
Software, algorithm	HB_frequency_plot.py	This paper		Source code file
Software, algorithm	AV_conductivity_plot.py	This paper		Source code file
Software, algorithm	AV_conductivity.py	This paper		Source code file

### Bioluminescent reporter assays for Ca^2+^ and cAMP

The intracellular cAMP and Ca^2+^ levels in rhodopsin-expressing HEK293S cells (human embryonic kidney 293 S cells, provided by Dr. Jeremy Nathans of Johns Hopkins University) were measured using the GloSensor cAMP assay and the aequorin assay, respectively, as described previously (Bailes and Lucas, 2013). HEK293S cells have been confirmed to be free from mycoplasma contamination. The identity of HEK293S cells was confirmed by similarity to HEK293 and HEK293T cells through STR profiling, and by morphological observation of the cells. The pGloSensor-20F cAMP plasmid (Promega) was used for the GloSensor cAMP assay. The wild type aequorin obtained by introducing two reverse mutations into the plasmid [pcDNA3.1+/mit-2mutAEQ] (Addgene #45539) (de la Fuente et al., 2012) was used for the aequorin assay. The rhodopsin expression plasmids were constructed based on pCS2+ (see the Zebrafish section) and used for transfection. For Gαq inhibition, YM-254890 (FUJIFILM Wako Pure Chemical Corp., 257–00631, Osaka, Japan) was added (1 μM) 5 min before the measurement. Green (500 nm) and violet (410 nm) LED lights were applied for 5 s in the GloSensor cAMP assay and for 1 s in the aequorin assay as light stimuli. Dual Head LED Light 505 nm (GB Life Science) and SPL-25-CC (REVOX, Inc) were used for green and violet LED light stimulation, respectively.

### Zebrafish

All transgenic zebrafish lines in this study were generated using the *mitfa^w2/w2^* mutant (also known as *nacre*) line, which lacks melanophores (Lister et al., 1999). To generate plasmids for transgenesis expressing optogenetic tools, the open reading frames (ORFs) of jumping spider (*Hasarius adansoni*) Rh1 (SpiRh1) (Koyanagi et al., 2008; Nagata et al., 2012), SpiRh1 S186F (Nagata et al., 2019), mosquito (*Anopheles stephensi*) Opn3 (Koyanagi et al., 2013), pufferfish (*Takifugu rubripes*) TMT opsin (Koyanagi et al., 2013), lamprey (*Lethenteron camtschaticum*) parapinopsin (Koyanagi et al., 2004), zebrafish (*Darnio rerio*) parapinopsin 1 and 2 (*parapinopsina* and *parapinopsinb* in ZFIN: https://zfin.org) (Koyanagi et al., 2015), honeybee (*Apis cerana*) UV and blue opsins (Terakita et al., 2008) or butterfly (*Papilio xuthus*) PxRh3 (Saito et al., 2019) were amplified by PCR and subcloned to pCS2+ (pCS2 +opto tool) containing a Flag tag sequence, a 2 A peptide sequence (P2A) from porcine teschovirus (PTV-1) (Provost et al., 2007; Tanabe et al., 2010), and TagCFP (Everon). For *Gt*ACR1 and ChrimsonR, GtACR-EYFP and ChrimsonR-tdTomato cDNAs were amplified from pTol1-UAS:ChrimsonR-tdTomato (Antinucci et al., 2020) and pFUGW-h*Gt*ACR1-EYFP (Govorunova et al., 2015), respectively, and subcloned to pCS2+. pENTR L1-R5 entry vectors containing five repeats of the upstream activation sequence (UAS) and the *hsp70l* promoter (Muto et al., 2017), and pENTR L5-L2 vectors containing the ORF of the optogenetic tools and the polyadenylation site of SV40 (SV40pAS) from pCS2 +were generated by the BP reaction of the Gateway system. The UAS-hsp70l promoter (Muto et al., 2017) and optogenetic tool expression cassettes were subcloned to the Tol2 donor vector pBleeding Heart (pBH)-R1-R2 (Dohaku et al., 2019), which was modified from pBH-R4-R2 and contains mCherry cDNA and SV40 pAS under the *myosin, light chain 7, regulatory* (*myl7*) promoter (van Ham et al., 2010) by the LR reaction of the Gateway system. To make the Tol2 vector to express GAL4FF (a modified form of the yeast transcription factor GAL4) in the heart, an about 900 bp fragment of the promoter and a 5′ untranslated region (UTR) of *myl7* (from pKHR7) (Hoshijima et al., 2016), GAL4FF cDNA (Asakawa et al., 2008), and SV40pAS were subcloned to a Tol2 vector pT2ALR-Dest (Dohaku et al., 2019) by the Gateway system. To make Tg fish, 25 pg of the Tol2 plasmids and 25 pg of transposase-capped and polyadenylated RNA were injected into one-cell-stage embryos. The *Tg(UAS:opto-tool)* fish that expressed the optogenetic tools in a GAL4-dependent manner were crossed with *TgBAC(vsx2:GAL4FF);Tg(UAS:RFP)* (Kimura et al., 2013), *Tg(myl7:GAL4FF)*, or *Tg(elavl3:GAL4-VP16)* (Kimura et al., 2013) to express the tools in hindbrain reticulospinal V2a neurons, cardiomyocytes, and all postmitotic neurons, respectively. *Tg(UAS:ChRWR-EGFP)* was used as a positive control (Kimura et al., 2013). For Ca^2+^ imaging, *Tg(5xUAS-hsp70l:GCaMP6s)* (Muto et al., 2017) was used. Adult zebrafish were raised at 28.5 °C with a 14 h light and 10 hr dark cycle. Individual larvae used for behavioral experiments were kept in the dark except for the observation of fluorescence and light exposure experiments.

### Immunostaining

For immunostaining, anti-Flag antibody (1:500, mouse, Sigma-Aldrich, St. Louis, MO, USA, Cat# F3165; RRID:AB_259529), anti-Myc tag (MT) antibody (1:500, mouse, Santa Cruz Biotechnology, Dallas, TX, USA, Cat# sc-40; RRID:AB_627268), anti-GFP (1:500, rat, Nacalai Tesque, Inc, Kyoto, Japan, Cat# 04404–84; RRID:AB_10013361), and anti-DsRed (1:500, rabbit, Takara Bio, Shiga, Japan, Cat# 632496; RRID:AB_10013483) antibodies were used as primary antibodies. CF488A anti-mouse IgG (1:500, H+L, Biotium, Inc, Fremont, CA, USA, Cat# 20018; RRID:AB_10557263), CF488A anti-rat IgG (1:500, H+L, Biotium, Inc, Cat# 20023; RRID:AB_10557403) and CF568 anti-rabbit IgG (1:500, H+L, Biotium, Inc, Cat# 20103; RRID:AB_10558012) antibodies were used as secondary antibodies. Individual fish were placed in 1.5 mL Eppendorf tubes and fixed in 4% paraformaldehyde in PBS at 4 °C for 1 hr. The fixed samples were washed three times with PBST, treated with acetone for 12 min at room temperature, washed again three times with PBST and twice with PBS-DT. The solution was replaced with 5% goat serum in PBS-DT and was kept at room temperature for 1 hr for blocking. Primary antibody was added to 5% goat serum in PBS-DT to achieve the dilution factor described above and incubated overnight at 4 °C. The samples were washed with PBS-DT six times for 15 min each wash. The incubation in secondary antibody solution, 5% goat serum in PBS-DT with the above-mentioned dilution factor, was performed overnight at 4 °C in the dark. After six washes of 15 min each in PBS-DT, the larvae were embedded in 1.5% agarose (low gelling temperature Type VII-A A0701, Sigma-Aldrich). Images were acquired using a confocal laser inverted microscope LSM700 (Carl Zeiss, Oberkochen, Germany). When acquiring images, the laser intensity was not changed by more than a factor of 2.

### Locomotion assay

3-dpf Tg larvae were quickly anesthetized with about 0.04% tricaine methanesulfonate (Nacalai Tesque, Inc, Kyoto, Japan, Cat# 01916–32) and embedded in 2.5% agarose in 1/10 Evans solution (134 mM NaCl, 2.9 mM KCl, 2.1 mM CaCl_2_, 1.2 mM MgCl_2_, and 10 mM Hepes; pH 7.8). The tail was set free by cutting the agarose around it. The agarose containing the embedded individual fish was placed in a 90 mm Petri dish filled with rearing water and kept under the microscope for 20 min to recover from anesthesia and to get used to the experimental environment which was followed by the first light exposure. For light stimulation, a patterned LED illuminator system LEOPARD (OPTO-LINE, Inc, Saitama, Japan) and the control software PolyScan2 (Mightex, Toronto, Canada) was used. LEDs with wavelengths of 405, 470, 520, and 620 nm, which are the closest values to the maximum absorption wavelength of each optogenetic tool, were used. The irradiation area was 0.30 mm × 0.34 mm in the hindbrain (Figure 2A). Tail movements were captured by an infrared CMOS camera (67 fps, GZL-C1L-41C6M-C, Teledyne FLIR LLC, Wilsonville, USA) mounted under the stage and StreamPix7 software (NorPix, Inc, Montreal, Canada) and analyzed by Tracker Video Analysis and Modeling Tool for Physics Education version 5.1.5. The timing of tail movement capture and light application of the reticulospinal V2a neurons was controlled by a USB DAQ device (USB-6008, National Instruments, Austin, TX, USA) and programming software (LabVIEW, 2015, National Instruments). The irradiation stimulation was repeated six times every 10 or 20 min for 1 s for G-protein-coupled rhodopsins, or 100 ms or 1 s for ChRWR with a minimum of eight individuals for each strain. The start and end times of tail movements were measured visually by StreamPix7 after the end of each trial. Trials in which swimming behavior was induced within 8 s after light stimulation were defined as induced trials. The percentage of induced trials was defined as locomotion rate, excluding trials in which swimming behavior was elicited before light stimulation. The time from the start of light application to the first tail movement was defined as latency, and the time from the start of the first tail movement to the end of that movement was defined as duration. The maximum distance the tail moved from the midline divided by the body length was defined as strength. To examine the tools’ activity in the inhibition of locomotion, 4-dpf Tg larvae were pretreated with 15 mM pentylenetetrazol (Sigma-Aldrich, Cat# P6500) and spontaneous tail movements were induced by white LED light (peak 640  nm; Kingbright Electronic Co., Ltd., New Taipei City, Taiwan) powered by a DC power supply (E3631A; Agilent Technologies, Santa Clara, CA, USA) for 5 s. After 500 ms from the onset of white LED light, hindbrain reticulospinal V2a was stimulated with the patterned LED illuminator. Trials in which swimming behavior stopped within 1 s after light stimulation were defined as locomotion-inhibition trials. The percentage of locomotion-inhibition trials was calculated and indicated in Table 1. Graphs were created with GraphPad Prism5 software (GraphPad Software, San Diego, CA, USA). All movies were created with VSDC Free Video Editor software version 6.4.7.155 (FLASH-INTEGRO LLC, Moscow, Russia) and Microsoft Movies & TV (Microsoft Corp., Redmond, WA, USA).

### Heartbeat experiments and heart size measurements

4-dpf Tg larvae were quickly anesthetized with about 0.2% tricaine methanesulfonate and embedded in 4% agarose in 1/10 Evans solution. Larvae embedded in agarose were placed in a 90 mm Petri dish filled with water and kept under a microscope for 20 min for recovery from anesthesia. Light stimulation was performed as described in the section of the locomotion assay. The area of irradiation was 0.17 mm × 0.25 mm, including the heart. The heart area in the Tg fish expressing MosOpn3, PufTMT, or LamPP was irradiated for 1 s with light wavelength of 520, 470, and 405 nm, respectively, which are the closest values to the maximum absorption wavelength of each optogenetic tool. The HBs of larvae were captured by an infrared CMOS camera (67 fps) and recorded with StreamPix7, as described above. The irradiation trial was repeated six times every 10 min for one fish and a total of four larvae were analyzed for each strain. The video recordings of HBs were observed using QuickTime player version 10.5 (Apple Inc, Cupertino, CA, USA). After opening videos with Fiji/ImageJ (National Institutes of Health, Bethesda, MD, USA) or Bonsai (Lopes et al., 2015), the entire heart was manually set as the region of interest (ROI), the luminosity (AU: arbitrary units) data in the ROI was used to create graphs of HBs using ggplot2 version 3.2.0 of R. As previously reported (Matsuda et al., 2017), the change in luminosity reflects the HB. To calculate the relative HB frequency, temporal changes in luminosity were obtained from the video using Bosai (Lopes et al., 2015) and the frames where HBs occurred were identified by the code (HB_frequency.py) created in Python ver. 3.5.6 (Python Software Foundation, Wilmington, DE, USA). The relative HB frequency was calculated from the HB frame data, 500 ms before and after each time point using Excel (Microsoft). Graphs of the average of relative HB frequency were created by ggplot2 in R or the code (HB_frequency_plot.py) in Python. The latency to cardiac arrest and the time to first resumption of HB were also measured. Graphs were created with GraphPad Prism5 software. All movies were created with VSDC Free Video Editor software. Simple HB experiments were also performed using a light source equipped with an MZ16 FA microscope and CFP (excitation light: 426–446 nm), GFP (460–500 nm), YFP (490–510 nm), and DSR filters (530–560 nm, Leica, Wetzlar, Germany), as indicated in Table 1. To measure the size of the heart, image data of the heart region was captured from videos in Fiji/ImageJ. The entire heart was manually identified, and its area was measured. For stimulation of MosOpn3, *Gt*ACR1-EYFP and ChrimsonR-tdTomato, a fluorescence detection filter (excitation 530–550 nm, emission 575IF nm, U-MWIG3, Olympus) was used.

### Analysis of time difference between atrial and ventricular contractions

The video recordings of HBs were analyzed by Bonsai (Lopes et al., 2015). Arbitrary positions of the atrium and ventricle were set as the ROIs. The luminosity data extracted from these ROIs was used to create graphs of HBs for both the atrium and ventricle using the code (AV_conductivity_plot.py) created in Python. The time difference between atrial and ventricular contractions was computed as the interval between the peak of the atrial HB and the corresponding peak of the ventricular HB using the code (AV_conductivity.py) in Python. HBs were considered undetected and thus excluded if the difference in AV contraction exceeded 0.5 s.

### Treatment with pertussis toxin (PTX) or BaCl_2_

For PTX treatment, after the irradiation trial was repeated three times, the larvae were removed from agarose then immersed in a solution containing PTX (0.2 µg/mL, Fujifilm Wako Pure Chemical Corp., Cat# 168–22471) for 3 min. After PTX treatment, larvae were embedded in agarose and placed on a Petri dish filled with deionized water. After larvae were kept in the Petri dish for 5 min, the heart area was irradiated three times every 10 min for 1 s (Figure 6). For control experiments of the PTX treatment, larvae were immersed in water instead of PTX solution for 3 min. For the BaCl_2_ treatment, 4-dpf larvae were embedded in agarose and placed in a Petri dish filled with water. After the irradiation trial was repeated twice, the water in the Petri dish was replaced with 1 mM BaCl_2_ (Fujifilm Wako Pure Chemical Corp., Cat# 025–00172) solution. After larvae were kept in this solution for 15 min, the heart area was irradiated three times every 10 min for 1 s (Figure 6). For control experiments of the BaCl_2_ treatment, larvae were kept in water instead of BaCl_2_. After opening videos with QuickTime Player, cardiac arrest time was measured. Cardiac arrest ratio was calculated as the ratio to cardiac arrest time in trial 1, and plotted as a graph using ggplot2 of R.

### Ca^2+^ live imaging

Tg larvae expressing GCaMP6s with or without the opto-tool in reticulospinal V2a neurons or cardiomyocytes were quickly anesthetized with 0.04% tricaine methanesulfonate and embedded in 4% agarose in 1/10 Evans solution. A 130 W light source (U-HGLGPS, Olympus, Tokyo, Japan) with a fluorescence detection filter (excitation 470–495 nm, emission 510–550 nm, U-MNIBA3, Olympus) was used to observe the fluorescence of GCaMP6s. The same filter set was used to stimulate SpiRh1, MosOpn3, PufTMT, and LamPP. For Tg larvae expressing SpiRh1[S186F] or LamPP, the reticulospinal V2a neurons or the heart area were irradiated with 405 nm for 1 s with the patterned LED illuminator system. A CCD camera (ORCA-R2, Hamamatsu Photonics, Shizuoka, Japan) attached to the microscope was used to capture the GCaMP6s fluorescence images at 9 fps. After image acquisition of V2a neurons, the high intensity region from the hindbrain to the spinal cord was set as the ROI using Fiji/ImageJ, and fluorescence intensity was measured. The relative change in fluorescence intensity (ΔF/F) was calculated by dividing the fluorescence intensity at each time point by the fluorescence intensity at the start of light stimulation for SpiRh1 or before stimulation (base line) for SpiRh1[S186F]. Graphs were created with GraphPad Prism5 software. After image acquisition for cardiomyocytes, videos of the heart were opened with Fiji/ImageJ, ROIs for the ventricle and atrium were set, and luminosity data were acquired. ΔF/F was calculated by dividing the fluorescence intensity at each time point by fluorescence intensity at the start of light stimulation for MosOpn3 and PufTMT, or by fluorescence intensity at the steady state (after HB resumption) for LamPP.

### Statistical analysis

Data were analyzed using R software package (versions 3.6.1 and 4.2.1). Statistical tests were applied as indicated in figure legends. A p-value of 0.05 or higher indicated a non-significant result. All data in the text and figures are expressed as the mean ± standard error of the mean (SEM). Linear mixed-effects model was applied using R package ‘nlme’ version 1.3–162.

## Data Availability

All data generated or analyzed during this study are included in the manuscript and supporting files and source data files have been provided for Figures 1-6.

## Additional files

MDAR checklist

Source code 1. Software that detects the timing of each heartbeat from the data of luminosity changes over time.

Source code 2. Software that calculates the average and SEM of heartbeats from the heartbeat data and plots them on a graph with time on the x-axis and heart rate on the y-axis.

Source code 3. Software that plots the temporal changes in luminosity data from any arbitrary position in the atrium and ventricle as waveform data. Within a specified time frame, it normalizes the data so that the maximum and minimum luminosity values are the same for both the atrium and ventricle.

Source code 4. Software that computes the interval between the peak of the atrial heartbeat and the corresponding peak of the ventricular heartbeat from the waveform data of the atrium and ventricle, as the time difference between atrial and ventricular contractions.
