# Supplementary material for: Optogenetic manipulation of Gq- and Gi/o-coupled receptor signaling in neurons and heart muscle cells
Source: eLife. 2023 Aug 17;12:e83974. doi: 10.7554/eLife.83974 (PMC10435233; doi:10.7554/eLife.83974)
Supplement: Peer review history

# Editor report: Editor's evaluation

**DOI:** 10.7554/eLife.83974.sa0

**Authors:** Suresh Jesuthasan

**Affiliations:** https://ror.org/02e7b5302 Nanyang Technological University Singapore

This work provides a valuable resource for scientists who wish to use optogenetics to manipulate GPCR signalling in larval zebrafish. It compares the physiological effects of vertebrate and invertebrate bistable rhodopsins, by expressing them in reticulospinal neurons or cardiomyocytes. The evidence for light–induced effects is solid, although some aspects of heart physiology have not been examined, in part due to technical limitations. Overall, the tools described here should be of interest to investigators working in a variety of areas.

# Decision letter

**DOI:** 10.7554/eLife.83974.sa1

**Affiliations:** https://ror.org/02e7b5302 Nanyang Technological University Singapore

Our editorial process produces two outputs: (i) public reviews designed to be posted alongside the preprint for the benefit of readers; (ii) feedback on the manuscript for the authors, including requests for revisions, shown below. We also include an acceptance summary that explains what the editors found interesting or important about the work.

**Decision letter after peer review:**

Thank you for submitting your article "Optogenetic manipulation of Gq–and Gi/o–coupled receptor signaling in neurons and heart muscle cells" for consideration by *eLife*. Your article has been reviewed by 2 peer reviewers, one of whom is a member of our Board of Reviewing Editors, and the evaluation has been overseen by Didier Stainier as the Senior Editor. The reviewers have opted to remain anonymous.

The reviewers have discussed their reviews with one another, and the Reviewing Editor has drafted this to help you prepare a revised submission.

Essential revisions:

1. More in–depth analysis that better captures the effects of manipulating GPCR signalling. For cardiac manipulation, this should include analyses of subtle changes in heart rate and contraction force. This is because modulation of cardiac activity (e.g. positive or negative chronotropic, inotropic, dromotropic, bathmotropic, and/or lusitropic responses) would represent better the physiological modulation of the heart via GPCR and downstream signaling events. In addition, different light intensities should be examined, as a light titration of observed effects could provide more meaningful insight into both rhodopsin responses and signaling mechanisms.

2. Figures should indicate when repeated measures are made on the same animal. This should be reflected in the statistical analysis. The effect of the trial number should be indicated. Additionally, the text should provide a quantitative description of the effects and statistical analysis.

3. Please provide better justification for the choice of different rhodopsins and sites of expression. For example, why were the rhodopsins tested in the reticulospinal V2a neurons selectively?

4. Please provide proper citations of relevant work in the field. This means citing all relevant papers, including those on cardiomyocytes. These papers should be discussed relative to the findings of the present work.

5. Address the issue of promiscuity of signalling via GPCRs.

*Reviewer #1 (Recommendations for the authors):*

1. The conclusion on page 9, that "these Flag–tagged G protein–coupled rhodopsins can be used for optogenetic manipulation of Gq– and Gi/o–mediated signaling in vertebrate cells" is strictly not true. The conclusion is valid for the cell type used. Would all vertebrate cells have the same properties as HEK cells? Which invertebrate cells do not?

2. The sentence in the middle of page 9 has a number of minor errors: It should be "...has been used to control GPCR signaling, but achieving temporally and spatially precise control has been difficult".

3. It should be noted that bistable rhodopsins have been used successfully in mice, e.g. see Mahn et al. 2021, as cited in the text. Thus, the third sentence in the section "Utility of bistable rhodopsin to study cell and tissue functions" on page 21, that states "It is most likely that when they are expressed in mammalian tissues, they can be used to optogenetically manipulate GPCR signaling in vivo". is incorrect. The experiment has already been done and reported.

4. The second sentence in the abstract is not correct. The tools were developed elsewhere, and this paper tests whether they function in two cell types in the zebrafish larva.

*Reviewer #2 (Recommendations for the authors):*

– The abstract implies that different tools were tested in neurons and cardiomyocytes. Only later it becomes obvious that both Gi and Gq pathways were optogenetically targeted in both cell types.

– The last sentence of the abstract could be more specific.

– The correct abbreviation for metabotropic receptors is mGluRs

– Activation of muscarinic M2 receptors reduces heart rate and contraction force but does not inhibit cardiac function under physiological conditions.

– Introduction of rhodopsins and retinal are kept too general. The same applies to the introduction of G protein activation upon retinal isomerization.

– Please only state the necessary amount of your own references after single statements, and if possible, please include supporting references by other teams as well. A more balanced way of citing will strengthen the meaningfulness of statements.

– In the introduction, the choice of described and later on tested rhodopsins is not clearly described.

– Papers by Copits et al., 2021 and Mahn et al., 2021 are cited in the introduction; but they are not sufficiently described. Accordingly, the observed data is not compared to the previously published data in the discussion. A detailed comparison of findings would help the reader to understand common mechanisms and to understand cell–type–specific findings.

– Please include quantitative data descriptions/numerical values in the results part, e.g. add percentages or values to statements indicating changes (reduction/increase, etc.).

– For experiments shown in Figures 2 and 3, a titration of light intensity would have been very useful.

– It is not clear why some rhodopsins showed mosaic expression whereas others were uniformly expressed. If this is an intrinsic characteristic of the Tg lines, it is difficult to compare any functional behavior.

– Please avoid data interpretation in the result part.

– Suppression of neuronal activity. You may want to be more specific.

– Description of Figure 5. Please be more specific with time points and indicate values for each level.

– The interpretation of light adaptation effects during prolonged illumination remains difficult. How can you dissect the effects of rhodopsin inactivation from cell–intrinsic compensatory mechanisms?

– Figure 3, 5, and throughout: Please try to perform experiments in the same manner for all tools compared. Please use graphs at the same time scales for all rhodopsins compared.

– From the provided data, you may not conclude that LamPP is switchable rhodopsin (which has been shown before). However, you can show that downstream signaling can be switched.

– Please never use statements such as "tended to weaken". Either an effect is statistically significant (then please state so) or no difference can be observed.

– The explanation of the effects of using repetitive light stimulation is not clear.

– To my knowledge, BaCl2 is not specific to inward rectifying K channels.

– Please do not comment on data not shown. You may want to consider including this data.

– You may want to consider further describing differences/similarities in downstream effects between dendritic, somatic, and axonal/presynaptic stimulation. This may help to dissect the molecular mechanism.

– It is not clear why SpiRh1 did not show effects in cardiomyocytes, especially taking into account recent studies using Neuropsin.

– The argument of cooperation of action potentials and ca^2+^ is difficult to follow.

– Why did Gi rhodopsins not change tail movement? Did you also look at the speed and extent of tail movement? Did you record neuronal activity? Which downstream signaling molecules might be missing in this specific neuronal cell type?

– Wavelength "were close/were apart" to/from one another. Please state the numerical values of maxima. Also, you may want to link to papers with spectra included, as the spectral overlap is most important. Is this a limitation of the available LEDs or actually (biologically) impossible to selectively activate or inactivate those rhodopsins?

– Can the very different kinetics of the effects in neurons vs cardiomyocytes be explained by photoreceptor properties or the different kinetics of the downstream signaling pathways?

– How do you know that the findings are transferrable to mammalian systems? This also applies to the co–expression of rhodopsin and fluorescent protein.

– The choice of wavelength for each tool is not obvious. Please include the maxima of activation/deactivation in table 1.

– Why were fish transferred to dishes with deionized water?

– Why were fluorescence intensity values differently normalized for SpiRh1 and the mutant? The same applies to the Gi–coupled rhodopsins. Please use the same normalization throughout.

– Please explain "luminosity".

– Table 1: Why did you check for cardiac arrest when using SpiRh1? Did you assess an increase in rate/contraction force etc.?

– Table 1: It seems as if MosOpn3 may induce an effect in neurons. Was this effect significant?

– Figure 1: Please better label axes. The relative response is not sufficient, even if explained in the legend. You may want to add a sketch of the experimental design to facilitate the reader in understanding each experiment.

– Figure 4: Why did the HB frequency not go down to "zero"?

– Figure 5: Why do you observe almost continuous ca^2+^ oscillations despite cardiac arrest? What is the underlying mechanism?

Language:

– "Light irradiation" and especially "light illumination", you may want to rephrase; I usually use either "light application" or "illumination".

– Please only use the term "on the other hand" following "on the one hand".

– Please correct "encephalopsin" and "parapinopsin" in the introduction.

– What do you mean by "relatively close expression levels".

# Reply: Author response

**DOI:** 10.7554/eLife.83974.sa2

Essential revisions:

1. More in–depth analysis that better captures the effects of manipulating GPCR signalling. For cardiac manipulation, this should include analyses of subtle changes in heart rate and contraction force. This is because modulation of cardiac activity (e.g. positive or negative chronotropic, inotropic, dromotropic, bathmotropic, and/or lusitropic responses) would represent better the physiological modulation of the heart via GPCR and downstream signaling events. In addition, different light intensities should be examined, as a light titration of observed effects could provide more meaningful insight into both rhodopsin responses and signaling mechanisms.

In response to these comments, we conducted additional experiments in which we activated bistable rhodopsins in cardiomyocytes with light of various intensities (0.5, 0.2, and 0.05 mW/mm^2^) to better examine the photosensitivity of the G protein-coupled bistable rhodopsins. We analyzed cardiac arrest rate, latency to cardiac arrest, and time to resumption of heartbeats (HBs). The results of these experiments are shown in Figure 4 and Figure 4-supplement 2, 3 in the revised manuscript. We described the data on page 15, line 16-page 16, line 1, as follows.

To analyze the photosensitivity of Gi/o-coupled rhodopsins, we applied light of various intensities for 1 s and examine their effect on HBs (Figure 4-supplement 2). Cardiac arrest was induced and sustained for over 20 s after stimulation of MosOpn3 with 0.05 mW/mm^2^ light for 1 s. Photoactivation of PufTMT and LamPP at lower light intensities (0.2 or 0.05 mW/mm^2^) resulted in cardiac arrest, but faster HB recovery than stimulation with 0.5 mW/mm^2^ light (Figure 4-supplement 2). The data indicate that the ability of MosOpn3 to suppress HBs is more photosensitive than PufTMT and LamPP in the zebrafish heart. We further examined atrial-ventricular (AV) conductivity by measuring the time difference between atrial and ventricular contractions before and after light stimulation when HBs had slightly recovered. There was no significant difference in AV conductivity before and after light stimulation (Figure 4-supplement 3).

Furthermore, we examined heart contractility (or lusitropy) by comparing the heart’s morphology when arrested by three different rhodospins: Gi/o-coupled rhodopsin MosOpn3, cation channelrhodopsin (ChrimsonR), and anion channelrhodopsin *Gt*ACR1. Activation of ChrimsonR led to a contracted heart, while *Gt*ACR1 induced relaxation of the heart. When arrested by MosOpn3, the heart appeared larger than the contracted heart arrested by ChrimsonR, but similar in size to the relaxed heart arrested by *Gt*ACR1 (Figure 4G). This suggests that the cardiac arrest caused by MosOpn3 activation was not due to continuous contraction, rather by the loss of heart muscle excitation. We described these data on page 15, lines 7-16.

These data suggest that Gi/o-coupled rhodopsin effectively reduced heart rate without significantly changing conductivity in the AV node (chronotropic effects). However, when Gi/o rhodopsin was strongly activated, it led to cardiac arrest by inhibiting the excitatory-contraction (EC) coupling process. This is consistent with the relaxation of the heart and the decrease in intracellular ca^2+^ levels following light stimulation. Since our experiments involved stimulating the entire heart area, the possibility that Gi/o-coupled rhodopsins are also able to inhibit AV conductivity cannot be completely excluded. Electrophysiological experiments would be necessary to fully understand how Gi/o-coupled rhodopsins exert control over the heart, particularly in terms of their bathmotropic effect. Unfortunately, such electrophysiological techniques have not yet been established for studying the hearts of zebrafish larvae. Nonetheless, we firmly believe that our detailed analysis of heartbeats under different light intensities provides strong evidence for our conclusion that Gi/o-coupled rhodopsin suppresses cardiomyocyte activity by G protein-coupled inwardly rectifier K^+^ channel (GIRK)-mediated inhibition of heart muscle excitation. We discussed this issue on page 22, line 17-page 24, line 2 in the Discussion section.

2. Figures should indicate when repeated measures are made on the same animal. This should be reflected in the statistical analysis. The effect of the trial number should be indicated. Additionally, the text should provide a quantitative description of the effects and statistical analysis.

We analyzed the effects on the reticulospinal V2a neurons and cardiomyocytes separately in individual larvae and presented average data in graphs. The results of these analyses were presented in the graphs as Figure 3A-E, Figure 3-supplement 2, Figure 4D-F, H, and Figure 4-supplement 2B. Furthermore, we investigated the effect of trial number, showing the data in Figure 3-supplement 1 and Figure 4-supplement 1, and explaining the data on page 12, lines 19-20 and page 15, lines 2-4 in the Result section. In addition, we provided a quantitative description of the effects in the text. Since there are too many statistics-related data (*p*-values), we decided not to describe them in the text, limiting the descriptions to the Figure legends.

3. Please provide better justification for the choice of different rhodopsins and sites of expression. For example, why were the rhodopsins tested in the reticulospinal V2a neurons selectively?

We examined the effects of several bistable rhodopsins on both reticulospinal V2a neurons and cardiomyocytes. We observed noticeable effects of Gq-coupled rhodopsins on reticulospinal V2a neurons, but no significant effects of these rhodospins on cardiomyocytes. Similarly, we found effects of Gi/o-coupled rhodopsins on cardiomyocytes, but no significant effects of these rhodopsins on reticulospinal V2a neurons. These discrepancies could be attributed to differences in the target cells and experimental conditions, suggesting the need of further optimization. We described the data on page 13, lines 16-22 and page 16, lines 9-10 in the Result section and Table 1, and discussed the relationship between the activity of bistable rhodopsins and their effects on target cells on page 21, lines 6-15 and page 24, line 19-page 25, line 2 in the Discussion section of the revised manuscript.

4. Please provide proper citations of relevant work in the field. This means citing all relevant papers, including those on cardiomyocytes. These papers should be discussed relative to the findings of the present work.

We further cited additional papers specifically related to optogenetic control of cardiomyocyte functions and usage of optogenetic tools in zebrafish (*Antinucci et al., 2020; Arrenberg et al., 2010; Bernal Sierra et al., 2018; Nussinovitch and Gepstein, 2015; Vogt eta l., 2015; Umeda et al., 2013; Watanabe et al., 2017*). We also cited papers related to the use of bistable rhodopsins in controlling functions of neurons and cardiomyocytes (*Cokic et al., 2021; Dai et la., 2022; Koyanagi et al., 2022; Makowka et al., 2019; Wagdi et al., 2022*). If the reviewer is able to indicate any additional references of relevance that might be missing, we would be happy to accommodate these in a follow-up revision.

5. Address the issue of promiscuity of signalling via GPCRs.

Using the reviewer’s suggestion as a prompt, we discussed this issue on page 26, lines 5-12 in the Discussion section, as follows. Certain GPCRs that share ligands are known to activate multiple signaling pathways and confer diverse cellular responses. They can interact with multiple types of G proteins. For example, there are three types of adrenergic receptors (ARs), α1, α2, and β, which bind to Gq, Gi/o, and Gs, respectively (β2 and β3 also bind to Gi), and activate different downstream signaling pathways (*Hilger et al. , 2018; Pierce et al. , 2002; Rockman et al. , 2002; Rosenbaum et al. , 2009*). Using optogenetic techniques, it might be possible to distinguish the in vivo roles of these adrenergic receptors and other GPCRs.

Reviewer #1 (Recommendations for the authors):

1. The conclusion on page 9, that "these Flag–tagged G protein–coupled rhodopsins can be used for optogenetic manipulation of Gq– and Gi/o–mediated signaling in vertebrate cells" is strictly not true. The conclusion is valid for the cell type used. Would all vertebrate cells have the same properties as HEK cells? Which invertebrate cells do not?

To address this request, we modified this sentence to “these Flag-tagged G protein-coupled rhodopsins can be used for optogenetic manipulation of Gq- and Gi/o-mediated signaling in human HEK293S cells” on page 10, lines 8-9.

2. The sentence in the middle of page 9 has a number of minor errors: It should be "....has been used to control GPCR signaling, but achieving temporally and spatially precise control has been difficult".

We modified this sentence, adjusting it to the reviewer’s suggestion.

3. It should be noted that bistable rhodopsins have been used successfully in mice, e.g. see Mahn et al. 2021, as cited in the text. Thus, the third sentence in the section "Utility of bistable rhodopsin to study cell and tissue functions" on page 21, that states "It is most likely that when they are expressed in mammalian tissues, they can be used to optogenetically manipulate GPCR signaling in vivo". is incorrect. The experiment has already been done and reported.

Since we agree with the reviewer, we modified this sentence to: “Bistable rhodopsins were shown to be expressed in mammalian tissues and used to optogenetically manipulate GPCR signaling in vivo (*Copits et al. , 2021; Dai et al. , 2022; Mahn et al. , 2021; Makowka et al. , 2019; Rodgers et al. , 2021; Wagdi et al. , 2022*)” on page 27, lines 1-4.

4. The second sentence in the abstract is not correct. The tools were developed elsewhere, and this paper tests whether they function in two cell types in the zebrafish larva.

To address this inaccuracy, we modified this sentence to: “To analyze the functions of GPCR signaling, using zebrafish, we assessed the effectiveness of animal G protein-coupled bistable rhodopsins that can be controlled into active and inactive states by light irradiation.”

Reviewer #2 (Recommendations for the authors):

– The abstract implies that different tools were tested in neurons and cardiomyocytes. Only later it becomes obvious that both Gi and Gq pathways were optogenetically targeted in both cell types.

Due to word limits in the abstract, we are unable to explain the optogenetic targeting of both the Gi and Gq pathways. However, we modified the Result section to make this readily apparent.

– The last sentence of the abstract could be more specific.

We changed the last sentence in the revised manuscript to: “These data indicate that these rhodopsins are useful for optogenetic control of GPCR-mediated signaling in zebrafish neurons and cardiomyocytes.” We revised the abstract slightly to keep it within the limit of 150 words.

– The correct abbreviation for metabotropic receptors is mGluRs

We corrected and replaced “GRM” with “mGluR” throughout the manuscript.

– Activation of muscarinic M2 receptors reduces heart rate and contraction force but does not inhibit cardiac function under physiological conditions.

We changed this sentence to: “acetylcholine binds to and activates the Gi/o-coupled muscarinic M2 receptor, which reduces heart rate and contraction”.

– Introduction of rhodopsins and retinal are kept too general. The same applies to the introduction of G protein activation upon retinal isomerization.

This is collaborative work between zebrafish researchers and bistable rhodopsin researchers. We wanted to describe the diversity of GPCRs and G protein-coupled bistable rhodopsin, as well as to introduce and discuss the possibility that diverse GPCR functions can be regulated by bistable rhodopsins. Since there are no word limits, we would like to maintain this description. However, if the reviewer still feels that the general part of the introduction should be reduced, we can simplify the introduction in a follow-up revision step.

– Please only state the necessary amount of your own references after single statements, and if possible, please include supporting references by other teams as well. A more balanced way of citing will strengthen the meaningfulness of statements.

We made every effort to cite relevant papers from other research groups to the best of our ability. However, if the reviewer believes that our citations are insufficient or insufficiently inclusive, we are open to their suggestions and will address them accordingly.

– In the introduction, the choice of described and later on tested rhodopsins is not clearly described.

We analyzed the available animal bistable rhodopsins and listed them in Table 1. We would like to avoid detailing all the animal bistable rhodopsins that we analyzed in the Introduction section. Instead, we modified the final sentence to: “In this study, we examined the optogenetic activity of Gq and Gi/o-coupled animal bistable rhodopsins (listed in Table 1) by expressing them in hindbrain reticulospinal V2a neurons that drive locomotion and cardiomyocytes in zebrafish.”

– Papers by Copits et al., 2021 and Mahn et al., 2021 are cited in the introduction; but they are not sufficiently described. Accordingly, the observed data is not compared to the previously published data in the discussion. A detailed comparison of findings would help the reader to understand common mechanisms and to understand cell–type–specific findings.

We have explained these studies in more detail on page 13, lines 15-16 in the Result section and page 24, lines 8-11 in the Discussion section of the revised manuscript.

– Please include quantitative data descriptions/numerical values in the results part, e.g. add percentages or values to statements indicating changes (reduction/increase, etc.).

We have described quantitative data in the Result section.

– For experiments shown in Figures 2 and 3, a titration of light intensity would have been very useful.

We conducted new and additional experiments with light stimulation at various light intensities, both in the reticulospinal V2a neurons and in the heart experiments (Figure 3, Figure 3-supplement 1, 2, Figure 4-supplement 2, 3). We described the relevant data on page 12, line 20-page 13, line 4 and page 15, lines 16-22.

– It is not clear why some rhodopsins showed mosaic expression whereas others were uniformly expressed. If this is an intrinsic characteristic of the Tg lines, it is difficult to compare any functional behavior.

The reviewer makes an accurate observation. At present, we cannot control the mosaic state of Gal4-UAS-mediated transgene expression. Therefore, we selected fish with similar expression levels for our study. This information was included in the manuscript (page 12, lines 2-5).

– Please avoid data interpretation in the result part.

The final sentence in a paragraph of the Results section includes an interpretation to explain the content. We believe this is both necessary and unavoidable. Apart from that, we eliminated as much interpretation as possible from other parts of the Results section.

– Suppression of neuronal activity. You may want to be more specific.

We modified this to: “suppress neurotransmitter release”.

– Description of Figure 5. Please be more specific with time points and indicate values for each level.

We revised the description of the Figure 5 legend. If our explanation is inadequate or unclear, we are open to revising it based on the Reviewer’s suggestion.

– The interpretation of light adaptation effects during prolonged illumination remains difficult. How can you dissect the effects of rhodopsin inactivation from cell–intrinsic compensatory mechanisms?

To address this issue, we analyzed light adaptation of Gi/o-coupled rhodopsins by repeating prolonged stimulation with light of a wavelength that only activates bistable rhodopsin. The hearts of Tg larvae expressing MosOpn3 or LamPP were irradiated with 0.5 mW/mm^2^ light of 520 nm for MosOpn3 or 0.4 mW/mm^2^ light of 405 nm for LamPP for 80 s, using three trials at 20 min intervals. During photoactivation of MosOpn3, HBs recovered slightly after about 40 s in all trials. In contrast, HBs gradually recovered during photoactivation of LamPP (Figure 5-supplement 1). Thus, during prolonged light stimulation, MosOpn3 maintained its active state for a relatively long period, while LamPP transitioned to an inactive state more rapidly. We described the data on page 17, lines 12-20. The data suggest that despite the differences among the bistable rhodopsins, there are likely intrinsic light adaptation mechanisms that inactivate bistable rhodopsins other than the photo-dependent reversal from an active to an inactive form. We discussed this issue on page 25, lines 6-15 in the Discussion section.

– Figure 3, 5, and throughout: Please try to perform experiments in the same manner for all tools compared. Please use graphs at the same time scales for all rhodopsins compared.

Based on the reviewer’s suggestion, we attempted to perform experiments in the same manner for all tools. However, for ca^2+^ imaging, it was necessary to change the experimental system when the excitation light of GCaMP6 activated rhodopsin (SpiRh1 and MosOpn3) and when it did not (SpiRh[S186F] and LamPP). Consequently, the manner of data extraction changes. Accordingly, we modified the presentation of graphs in Figures 3 and 5. This was explained in the text and figure legends.

– From the provided data, you may not conclude that LamPP is switchable rhodopsin (which has been shown before). However, you can show that downstream signaling can be switched.

We modified the sentence to: “Therefore, the activity of LamPP can be turned on and off by using light of different wavelengths in the zebrafish heart” on page 17, lines 10-11.

– Please never use statements such as "tended to weaken". Either an effect is statistically significant (then please state so) or no difference can be observed.

We decided that for statements related to experimental results with no significant difference, we either stated that there was no significant difference, or we did not describe the difference.

– The explanation of the effects of using repetitive light stimulation is not clear.

We performed repetitive light stimulation of cardiomyocytes expressing MosOpn3, PufTMT, and LamPP every 10 min before and after PTX or BaCl2 treatment as shown in Figure 6A or 6E. Details of the experimental procedure are described in “Treatment with pertussis toxin (PTX) and BaCl_2_” of the Material and methods section, and in the Figure 6 legend.

– To my knowledge, BaCl2 is not specific to inward rectifying K channels.

According to literature, not only BaCl2 but other chemicals were used to block GIRKs. We did not analyze the specificity of BaCl2 to GIRKs in this study. We would like to maintain the assertion that the inhibitory experiments with BaCl_2_ suggest the possibility that the Gio rhodopsins might be acting through GIRKs.

– Please do not comment on data not shown. You may want to consider including this data.

Photostimulation of the hindbrain of Tg larvae expressing SpiRh1 in reticulospinal V2a neurons induced tail movements, suggesting that SpiRh1 functions in the axons of reticulospinal V2a neurons. However, we think that more robust experimental data are necessary to conclude this. Thus, we decided to delete this description.

– You may want to consider further describing differences/similarities in downstream effects between dendritic, somatic, and axonal/presynaptic stimulation. This may help to dissect the molecular mechanism.

We agree with the reviewer that it is extremely important to analyze differences and similarities in the effects of dendritic, somatic, and axonal/presynaptic stimulation, as was done in previous reports (Mahn et al., 2021; Copits et al., 2021). However, we spent considerable resources, time and effort to compare the activity of many bistable rhodopsins in two different systems, so we do not wish to complete such an analysis in this study.

– It is not clear why SpiRh1 did not show effects in cardiomyocytes, especially taking into account recent studies using Neuropsin.

We also do not know the reason. Given the differences in the rhodopsins used and their expression systems, no easy conclusions can be drawn. We cited a relevant paper and further discussed this issue on page 22, lines 6-15 in the revised manuscript.

– The argument of cooperation of action potentials and ca^2+^ is difficult to follow.

To assist with comprehension, we added the following sentence to page 21, line21-page 22, line 3. “While the depolarization signal directly participates in the information transmission of neural circuits, the increase in intracellular ca^2+^ may regulate changes in synaptic transmission efficiency by modifying neurotransmitter receptors and/or channels and controlling their function and localization”. If this is still insufficiently clear, we are open to making further revisions based on the reviewer’s specific suggestions.

– Why did Gi rhodopsins not change tail movement? Did you also look at the speed and extent of tail movement?

Although a slight functional inhibition of V2a neurons by MosOpn3 was observed, it was not statistically significant. While this was not mentioned in this paper (it is described in the paper that we submitted concurrently), experiments using the anion channel *Gt*ACR1 clearly showed inhibition of tail movements, in contrast with MosOpn3. We think that further examination of experimental conditions is necessary. We described this issue on page 13, lines 16-22.

Did you record neuronal activity? Which downstream signaling molecules might be missing in this specific neuronal cell type?

We believe that electrophysiological experiments are necessary to answer many of the questions raised in this study. However, in this manuscript, we did not perform electrophysiological experiments because we wanted to focus on the differences in bistable rhodopsin activity.

– Wavelength "were close/were apart" to/from one another. Please state the numerical values of maxima. Also, you may want to link to papers with spectra included, as the spectral overlap is most important. Is this a limitation of the available LEDs or actually (biologically) impossible to selectively activate or inactivate those rhodopsins?

We listed the wavelengths of light used in this study and the light for maximum activation or inhibition in Table 1 and offered an explanation in the table legend. As shown in Table 1, we used wavelengths that were close to those that result in maximum activation or inhibition. However, for some tools, we could only use wavelengths that were slightly apart from the optimum value due to the availability of LEDs.

– Can the very different kinetics of the effects in neurons vs cardiomyocytes be explained by photoreceptor properties or the different kinetics of the downstream signaling pathways?

At this point, both possibilities seem likely. However, we have no data to clarify this, so we decided not to mention it.

– How do you know that the findings are transferrable to mammalian systems? This also applies to the co–expression of rhodopsin and fluorescent protein.

We do not argue that these findings are transferrable to a mammalian system. However, data obtained from zebrafish frequently provide valuable insight for mammalian experiments, so there is no reason to refute this. Therefore, we would like to leave the statement that notes this possibility.

– The choice of wavelength for each tool is not obvious. Please include the maxima of activation/deactivation in table 1.

Where information was available, we incorporated light wavelengths for maximum activation and inactivation of bistable rhodopsins in Table 1.

– Why were fish transferred to dishes with deionized water?

In this experimental system, when treating inhibitors, small volumes of liquid were used, and photostimulation was performed in water, which has a relatively high volume, and since PTX is valuable, small volumes were used, and the experiment was performed in water after treatment. The BaCl_2_ treatment was performed in the same way to achieve equivalent conditions. We omitted the description of the BaCl_2_ treatment because it did not affect the interpretation of the data.

– Why were fluorescence intensity values differently normalized for SpiRh1 and the mutant? The same applies to the Gi–coupled rhodopsins. Please use the same normalization throughout.

In the original manuscript, we created expression data for the tool by incorporating raw data as much as possible. However, due to variations in staining conditions, we followed the reviewer’s suggestion and generated data by normalizing all samples as much as possible based on the control RFP (mCherry) expression in Figure 2B and 4A.

– Please explain "luminosity".

We explained the method to analyze heartbeats in the material and methods section as follows: “After opening videos with Fiji/ImageJ (National Institutes of Health, Bethesda, MD, USA), the entire heart was manually set as the region of interest (ROI), and the luminosity (AU: arbitrary units) data in the ROI was used to create graphs of HBs using ggplot2 version 3.2.0 of R. As previously reported (*Matsuda et al. , 2017*), the change in luminosity reflects the HB.” We also explained this in the legend of Figure 4.

– Table 1: Why did you check for cardiac arrest when using SpiRh1? Did you assess an increase in rate/contraction force etc.?

We examined heart rate broadly and found no significant differences with or without stimulation. In Table 1, we only showed the cardiac arrest ratio. We changed the sentence in the figure legend as follows: “Neither cardiac arrest, bradycardia, nor tachycardia was induced with either 490-510 nm, 530-560 nm (microscope-equipped light source, *n*=100), or 520 nm (LED, *n*=2) light.”

– Table 1: It seems as if MosOpn3 may induce an effect in neurons. Was this effect significant?

It was not significant. Thus, we decided not to describe this issue in the Result section.

– Figure 1: Please better label axes. The relative response is not sufficient, even if explained in the legend. You may want to add a sketch of the experimental design to facilitate the reader in understanding each experiment.

We created a schematic diagram to illustrate the cell culture experiments in Figure 1A. In addition, we included on the y-axis of Figure 1 “intracellular ca^2+^ concentration” (B, C, and E) and “intracellular cAMP concentration” (D).

– Figure 4: Why did the HB frequency not go down to "zero"?

Figure 4C shows the average of the heart rate data for six consecutive stimulation trials of four rhodopsin-expressing larvae. The cardiac arrest rate was determined by the ratio of trials in which the HBs stopped due to light stimulation. The latency to cardiac arrest and the time to resumption of HB varied between trials and individuals, and there were instances where a slight HB occurred at any given time. Since these instances were included in the calculation, the average of HB frequency was not zero.

– Figure 5: Why do you observe almost continuous ca^2+^ oscillations despite cardiac arrest? What is the underlying mechanism?

As above, Figure 5 shows the average GCaMP6s fluorescence of multiple larvae, and a low value of oscillation was observed when trials that did not stop completely were included.

Language:

– "Light irradiation" and especially "light illumination", you may want to rephrase; I usually use either "light application" or "illumination".

We changed “light irradiation” to “light application”.

– Please only use the term "on the other hand" following "on the one hand".

We rephrased “on the other hand” to “in contrast”.

– Please correct "encephalopsin" and "parapinopsin" in the introduction.

We corrected these terms.

– What do you mean by "relatively close expression levels".

The expression was misleading. We changed the sentence to: “we established multiple Tg lines and analyzed stable Tg lines (F_1_ or later generations) that expressed equally high – but varying – levels of these tools.”
